# Dynamic control of circumrotation of a [2]catenane by acid‐base switching

**DOI:** 10.1002/open.202300304

**Published:** 2024-02-09

**Authors:** Kelun Shi, Guohui Jia, Ying Wu, Shilong Zhang, Jiawen Chen

**Affiliations:** ^1^ SCNU-UG International Joint Laboratory of Molecular Science and Displays National Center for International Research on Green Optoelectronics Guangzhou 510006 P. R. China

**Keywords:** oligo ether-chain, self-complexes, catenane

## Abstract

Dynamic control of the motion in a catenane remains a big challenge as it requires precise design and sophisticated well‐organized structures. This paper reports the design and synthesis of a donor‐acceptor [2]catenane through mechanical interlocking, employing a crown ether featuring two dibenzylammonium salts on its side arms as the host and a cyclobis(paraquat‐*p*‐phenylene) (**CBPQT** ⋅ 4PF_6_) ring as the guest molecule. By addition of external acid or base, the catenane can form self‐complexed or decomplexed compounds to alter the cavity size of the crown ether ring, consequently affecting circumrotation rate of **CBPQT** ⋅ 4PF_6_ ring of the catenane. This study offers insights for the design and exploration of artificial molecular machines with intricate cascading responsive mechanisms.

## Graphical abstract

We reports a mechanical interlocking donor‐acceptor [2]catenane, which is able to form varying degrees of self‐complexed or decomplexed compounds that alter the cavity size of the crown ether and affecting the circumrotation behavior of **CBPQT** ⋅ 4PF_6_ ring of the catenane.

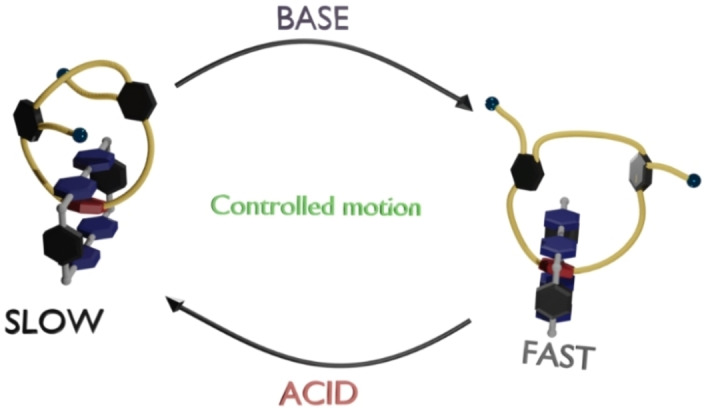

## Introduction

Catenane is one of the most important mechanically interlocked molecules (**MIMs**) as it consists of two or more interlocked ring units featuring with special topology.[^1^, ^2^, ^3^, ^4^, ^5^, ^6^] Since the pioneering works by Schill and Zürcher,^[7]^ the combination of catenanes and other supramolecular systems has led to a large varieties of spatial geometries that have even more complicated topological structures. Representative examples are carbon nano‐belt,^[8]^ molecular necklace,^[9]^ poly[n]catenanes,^[10]^ cages,^[11]^ bio‐mimic function of butterfly^[12]^ and molecular motors.[^13^, ^14^, ^15^, ^16^] In the past several decades, bistable catenanes have received many attentions as they have been reported for the applications of photocatalyst,^[17]^ molecular recognitions^[18]^ and ion sensing.[^19^, ^20^] The use of external stimulus to trigger and control the motions of certain component in such host‐guest systems has provided additional dynamic approach to the construction of muti‐functional nanoscale machines beyond their unique self‐locking features. By design, catenanes have been synthesized and showed controlled motion in the presence of light,[^21^, ^22^, ^23^] chemical stimulation,[^24^, ^25^, ^26^, ^27^, ^28^, ^29^] chemical fuels,[^30^, ^31^, ^32^] thermal^[33]^ and electricity[^16^, ^34^] with high efficiency.

Alternatively, another approach to dynamically control the motion of catenane can rely on the reconfiguration of the molecular machine by itself. This approach has been commonly found among the non‐catenane systems, for example, linear molecular rotaxanes are attached with additional functional groups, pro‐longing their constituent parts such as the axles, to raise the energy barrier and therefore controlling the transitional motion.[^35^, ^36^, ^37^, ^38^] However, catenane‐based supramolecular systems that undergo specific deformation of topologies, such as host cavities to influence the dynamic properties for guest shuttling or co‐conformational switching are more challenging than linear‐shaped rotaxanes, as catenanes are less likely to twist or fold and therefore have limited free spaces to reorientate themselves. Self‐complexing and self‐assembling systems have been shown to be a successful manner to control the relative motions of nano‐scale molecular devices.[^39^, ^40^, ^41^, ^42^, ^43^] Among the bistable molecular machine systems, the association between oligo‐ether chains and positive cations are widely used to establish the host‐guest interaction. The cavity of the crown ether can accommodate not only metal cations guest, but also organic molecular guests.[^43^, ^44^, ^45^, ^46^, ^47^, ^48^] Intermolecular complexation systems and self‐complexation systems based on dibenzylammonium salt/crown ether couples have been reported, and have been applied in the construction of light‐harvesting antenna,^[49]^ fluorescent sensing,^[50]^ nanofibers,^[51]^ information ratchet.^[36]^ The selective inclusion of guest compound to change the size of the cavity has been proved successfully to the capture and release of specific guest molecules.[^52^, ^53^, ^54^, ^55^, ^56^]

**Scheme 1:**
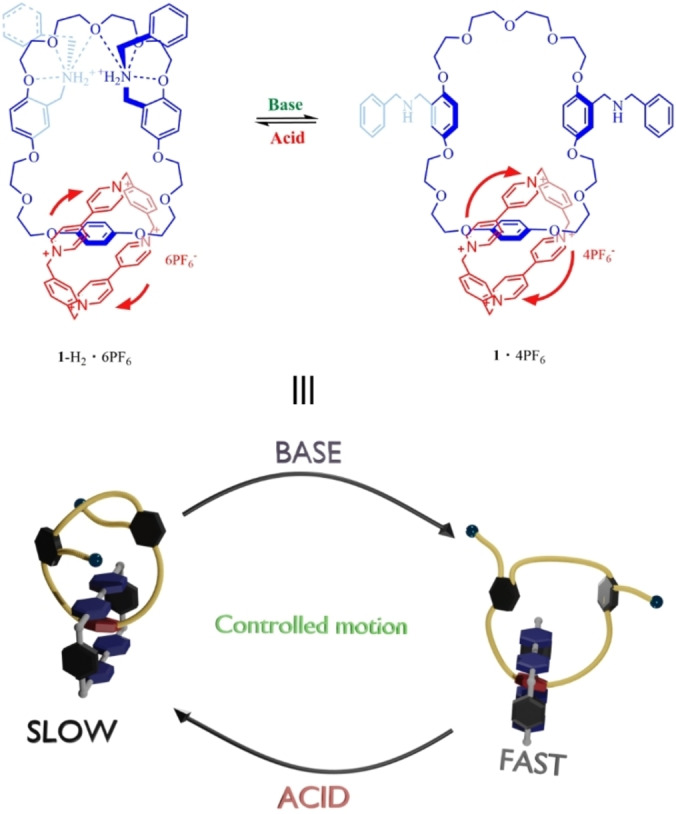
The structures of different states of [2]catenane with different circumrotation rate of **CBPQT** ⋅ 4PF_6_ ring.

In the present study, we report a new donor‐acceptor [2]catenane **1**‐H_2_ ⋅ 6PF_6_ that has inherent hots‐guest interaction to dynamic tune the cavity of crown ether and thereby affecting circumrotation rate of the ring. The catenane is formed by mechanical interlocking of the crown ether ring containing two dibenzylammonium salts on the side arms and a cyclobis(paraquat‐*p*‐phenylene) (**CBPQT** ⋅ 4PF_6_) ring (Scheme 1). By addition of external acid or base, the catenane complexes or de‐complexes with the dibenzylammonium salts to dynamically change the cavity of the crown ether accordingly. Modifications of the cavity size of crown ether therefore influences the circumrotation of **CBPQT** ⋅ 4PF_6_ in the catenane, which can be monitored by ^1^H NMR. When the two side arms are in protonated dibenzylammonium salts state (Scheme 1, **1**‐H_2_ ⋅ 6PF_6_), the cavity of the crown ether ring remains crowded. As a consequent, the circumrotation of **CBPQT** ⋅ 4PF_6_ is limited. When the dibenzylammonium salts are gradually deprotonated (Scheme 1, **1**‐H_1_ ⋅ 5PF_6_), the cavity size of the crown ether ring increases, enhancing the freedom of circumrotation of **CBPQT** ⋅ 4PF_6_. When the two dibenzylammonium salts are fully deprotonated (Scheme 1 **1** ⋅ 4PF_6_), it results in the freest circumrotation of **CBPQT** ⋅ 4PF_6_.

## Results and Discussion

### Molecular Design and Synthesis

**Scheme 2:**
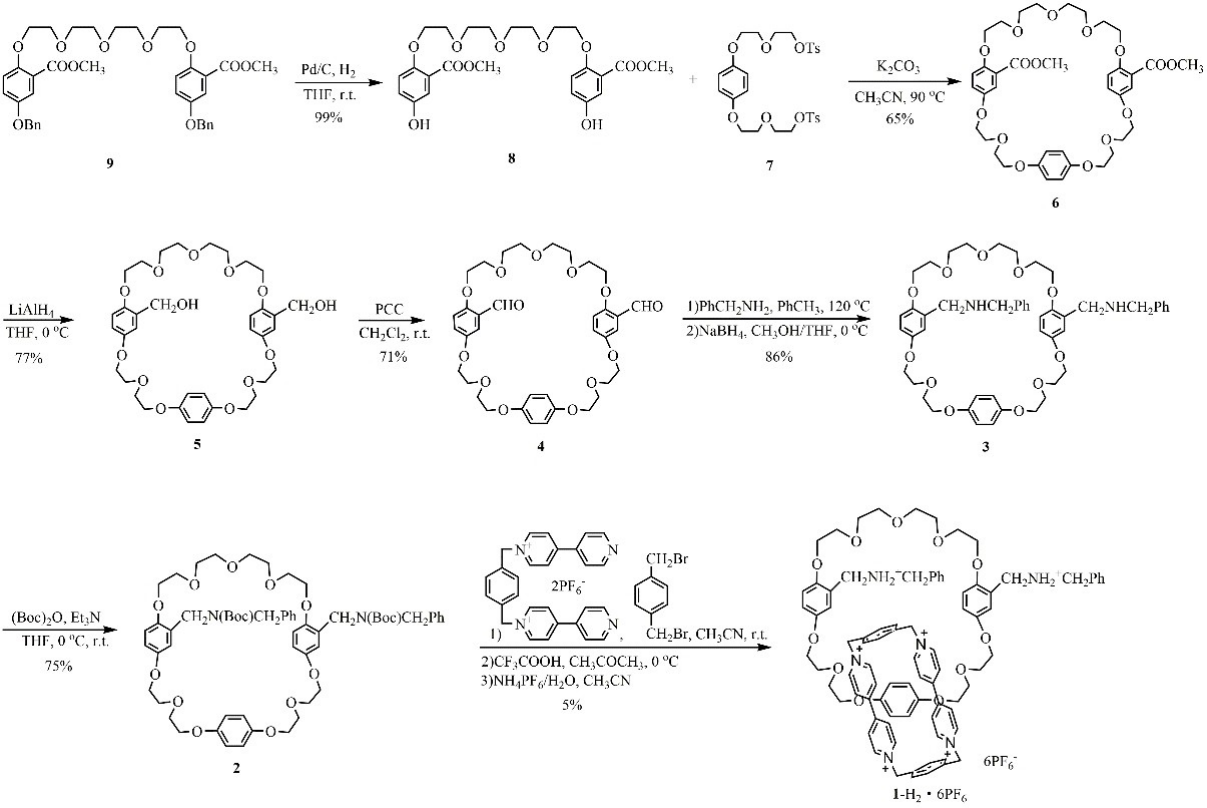
The synthetic route of [2]catenane **1**‐H_2_ ⋅ 6PF_6_.

The synthesis of [2]catenane **1**‐H_2_ ⋅ 6PF_6_ is outlined in Scheme 2. Compound **9**, obtained by the reported procedures,^[57]^ was placed under high‐pressure hydrogen atmosphere in the presence of Pd/C for 48 h at r.t., affording **8** in a quantitative yield. Subsequently, **8** and **7** were heated at 90 °C for 2 d to generate **6** as a tri‐*p*‐phenylene‐39‐crown‐11 (**TPP39C11**) derivatives bearing two ester substituents on two aromatic rings. After reduction by LiAlH_4_, the two methyl esters groups of **6** were converted into corresponding hydroxyl groups, giving rise to compound **5**. **5** was further oxidated under mild condition to generate di‐aldehyde compound **4**. Condensation of **4** resulted in **3** that is the crown ether with two dibenzylamines on its side arms. Subsequently, the amines were protected by (Boc)_2_O to afford **2**. The p‐xylylene‐bis(4‐(4‐pyridyl)pyridinium) bis(hexafluorophosphate) was then reacted with 1,4‐bis(bromomethyl)benzene in the presence of **2** for 15 d, and after deprotection by CF_3_COOH and counterion exchange, the target donor‐acceptor [2]catenane **1**‐H_2_ ⋅ 6PF_6_ was obtained.

### Structural Change of Crown Ether 3 by Acid‐Base Switching

**Figure 1:**
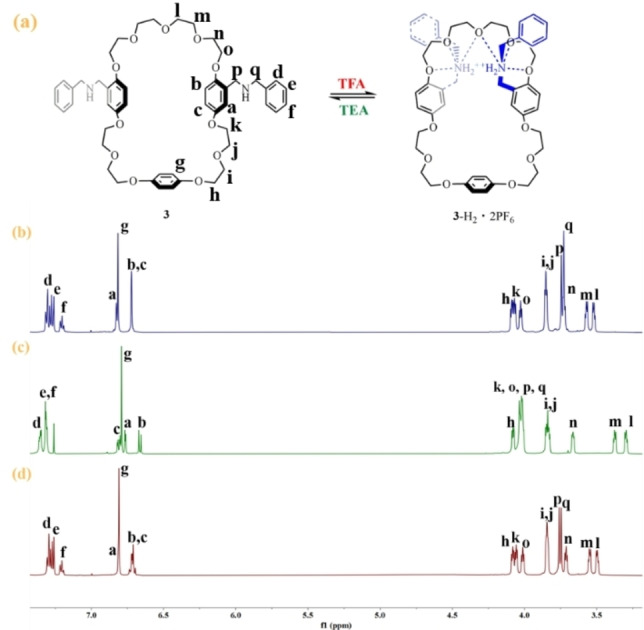
(a) Schematic representation of the changes of the cavity of crown ether **3** by acid‐base switching, (b) partial ^1^H NMR spectra (600 MHz, 298 K, CDCl_3_) of 11.53 x10^−6^ M compound **3**, (c) the solution obtained after addition of 3.0 equiv. of TFA to part (b), (d) the solution obtained after addition of 4.0 equiv. of TEA to part (c).

We first employed crown ether **3** as a control compound to understand the change of the cavity upon addition of corresponding acid or base. The acid‐base induced changes of structure of crown ether **3** were confirmed by ^1^H NMR spectroscopy. Figure 1b depicts the partial ^1^H NMR spectrum of compound **3**, and all the signals can be well assigned (Supporting information Figures S2–S9).

The signal of H_a_ is observed at 6.83 ppm, and H_p_ and H_q_ are found to be at 3.74 ppm and 3.73 ppm respectively (Figure 1b). Upon the addition of 3.0 equiv. TFA, H_a_ was found to have an up‐field shift to 6.77 ppm (Figure 1c), which is attributed to the aromatic shielding effect, indicating that H_a_ is situated inside the cavity of the crown ether ring moiety.^[58]^ The chemical shifts of H_p_ and H_q_ were observed to move down‐field, at 4.04 ppm and 4.02 ppm respectively. These shifts are attributed to the strong hydrogen bond interaction between the NH_2_^+^ groups and the oxygens of the crown ether when the protonated dibenzylammonium salts sit into the crown ether cavity.^[59]^ Additional evidence was based on the ^1^H‐^1^H NOESY spectrum which reveals the presence of NOE cross‐peaks (Supporting information Figure S18) between H_q_ and H_n_. Furthermore, we were delighted to find that the fully protonated crown ether forms a self‐complexed compound in an intramolecular manner, rather than an intermolecular manner which might result in a [c2]daisy chain or linear oligomer,[^60^, ^61^] as the proton signals of the ‐OCH_2_CH_2_O‐ fragment are distinctly separated into eight distinct sets (Figure 1b). With the addition of 4.0 equiv. TEA, H_a_ was found to shift down‐field, while H_p_ and H_q_ were observed to move up‐field, regaining the original spectrum (Figure 1d and 1 b), indicating that switching between crown ether **3** and **3**‐H_2_ ⋅ 2PF_6_ states is fully reversible, which sets a solid base for dynamical tuning of circumrotation of **1**.

We next tested the possibility whether only one NH group is protonated to NH_2_^+^ to form a monoprotonated self‐complexed compound (Supporting information Scheme S2). ^1^H NMR titration experiments were therefore performed. The 0.5 equiv. TFA was incrementally added to the solution of **3**, and H_a_ can serve as the probe to track the entire titration process. The results demonstrate that with the addition of TFA, H_a_ was found to continuously shift up‐field (Supporting information Figure S20c). However, no solid evidences were found to show the exclusive formation of monoprotonated species. After addition of 2.5 equiv. TFA, the crown ether **3** stays in the completely protonated state, as it is evident by the spectrum when compared to the spectrum of Figure 1c.

With the experimental results of **3** in hands, we then moved to test the behavior of **1**‐H_2_ ⋅ 6PF_6_. The UV‐vis absorption spectra of TEA titration of **1**‐H_2_ ⋅ 6PF_6_ were measured at first (Supporting information Figure S37). The results showed that the catenane **1**‐H_2_ ⋅ 6PF_6_ has the maximum absorption at 454 nm. With the addition of 0.5 equiv. TEA in portions, the peak gradually increased, indicating the process from self‐complexation to de‐complexation.[^33^, ^62^] Therefore, the cavity size of the crown ether ring gradually increases. When the system was treated with 3.0 equiv. TFA, the UV absorption spectra regained to its initial state (Supporting information Figure S37).

### Dynamic Control of Circumrotation of [2]Catenane 1‐H_2_ ⋅ 6PF_6_ by Acid‐Base Switching

**Figure 2:**
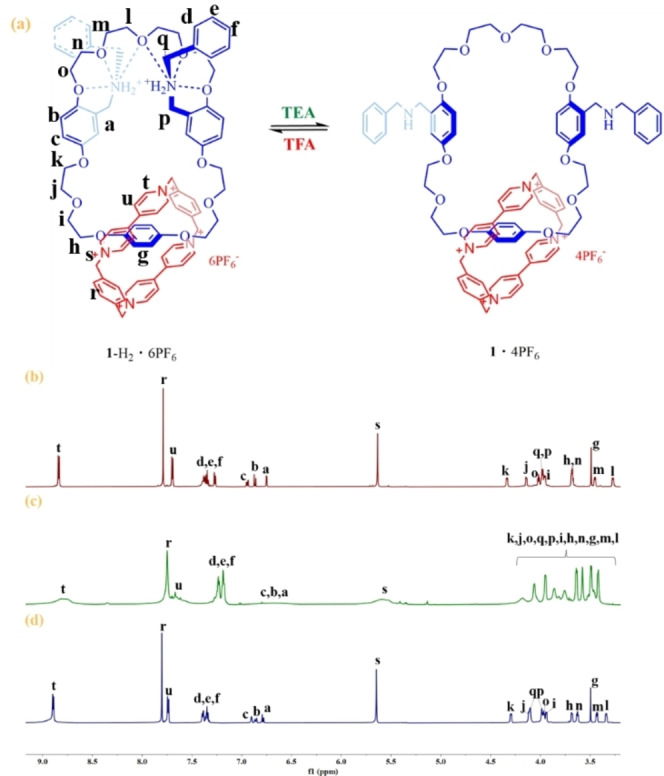
(a) Schematic representation of the changes of the cavity of catenane by acid‐base switching, (b) partial ^1^H NMR spectra (600 MHz, 298 K, CD_3_CN) of 7.52 x10^−6^ M **1**‐H_2_ ⋅ 6PF_6_, (c) the solution obtained after adding 3.0 equiv. of TEA to part (b), (d) the solution obtained after adding 6.0 equiv. of TFA to part (c).

^1^H NMR was employed to monitor the acid‐base switching process as well. Upon addition of 0.5 equiv. TEA to a solution of **1**‐H_2_ ⋅ 6PF_6_ in portions, the peak of H_a_ became broader and eventually overlaped with the peaks of H_b_ and H_c_ (Supporting information Figure S30c). After addition of 2.5 equiv. TEA, the catenane maintained at the state of full deprotonation, which is indicated by the spectrum when compared to Figure 2c. Subsequently, an excessive 6.0 equiv. TFA was added, and it was observed that the signals of protons were consistent with the initial **1**‐H_2_ ⋅ 6PF_6_ signals (Figure 2d and Figure 2b), indicating that the acid‐base switching between catenane **1**‐H_2_ ⋅ 6PF_6_ and **1** ⋅ 4PF_6_ states is reversible.

**Figure 3:**
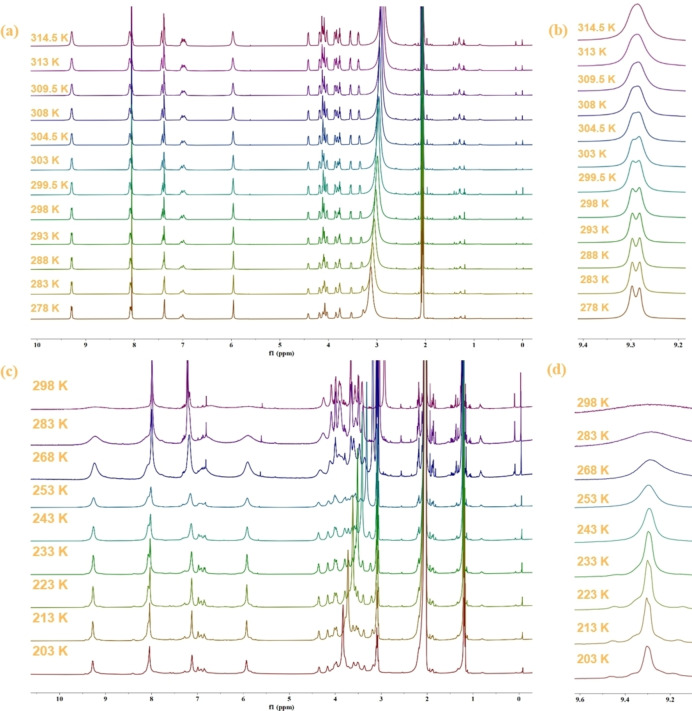
(a) VT ^1^H NMR spectra (400 MHz, CD_3_COCD_3_) of **1**‐H_2_ ⋅ 6PF_6_, (b) VT ^1^H NMR spectra of H_t_ of **1**‐H_2_ ⋅ 6PF_6_, (c) VT ^1^H NMR spectra (400 MHz, CD_3_COCD_3_) of **1** ⋅ 4PF_6_, (d) VT ^1^H NMR spectra of H_t_ of **1** ⋅ 4PF_6_.

The circumrotation rates of different states of catenane **1** were measured by variable temperature ^1^H NMR.^[63]^ Proton H_t_ was selected as the probe to monitor the spectral changes at different temperatures. Figure 3a shows partial ^1^H NMR spectra of **1**‐H_2_ ⋅ 6PF_6_ at different temperatures. As the temperature rises, the shape of H_t_ gradually becomes blunt (Figure 3b), slowly merging into a single peak, and coalesces to form a single peak at 309.5 K. Through simulation calculations (Supporting information Figures S32–S33), it is determined that the circumrotation rate of fully protonated **1**‐H_2_ ⋅ 6PF_6_ at 298 K is 4.77 s^−1^ and ▵G^≠^(298 K) is 16.52 kcal ⋅ mol^−1^.

Figure 3c displays the variable temperature ^1^H NMR spectra of **1** ⋅ 4PF_6_. It can be observed that H_t_ is a blunt broad peak at 298 K (Figure 3d). As the temperature decreases, the peak gradually becomes sharper and exhibits a tendency to split at 233 K. However, even when the temperature was further decreased to 203 K, the signal peak didn't fully split. It suggests that the circumrotation rate of catenane **1** ⋅ 4PF_6_ is relatively fast, much faster than that of catenane **1**‐H_2_ ⋅ 6PF_6_. Although absolute value of the circumrotation rate of catenane **1** ⋅ 4PF_6_ could not be determined, it is evident that the rate is much faster than that of catenane **1**‐H_2_ ⋅ 6PF_6_, indicating the successful modulation of circumrotation rate of catenane by acid‐base switching.

## Conclusions

A new donor‐acceptor [2]catenane **1**‐H_2_ ⋅ 6PF_6_ was designed and prepared by template synthesis approach. The crown ether containing two dibenzylammonium salts on the side arms are able to form to a self‐complexed or de‐complexed compound under different acid‐base conditions, thereby altering the cavity size of the crown ether ring and subsequently affecting the circumrotation motion of the **CBPQT** ⋅ 4PF_6_ ring within the catenane. It can be determined by variable temperature ^1^H NMR that the rotational rate of **1**‐H_2_ ⋅ 6PF_6_ is the slowest, and a much faster rate of **1** ⋅ 4PF_6_ could be observed when the system is fully deprotonated. This study offers a new insight for the design and exploration of complex artificial molecular machines with cascading and multiple responses.

## Supporting Information

The authors have cited additional references within the supporting information.

## Conflict of interests

The authors declare no conflict of interest.

1

## Supporting information

As a service to our authors and readers, this journal provides supporting information supplied by the authors. Such materials are peer reviewed and may be re‐organized for online delivery, but are not copy‐edited or typeset. Technical support issues arising from supporting information (other than missing files) should be addressed to the authors.

Supporting Information

## Data Availability

Research data are not shared.
