# Supplementary material for: Dynamic control of circumrotation of a [2]catenane by acid‐base switching
Source: ChemistryOpen. 2024 Feb 9;13(8):e202300304. doi: 10.1002/open.202300304 (PMC11319237; doi:10.1002/open.202300304)
Supplement: Supplementary file 1 — Supporting Information [file OPEN-13-e202300304-s001.pdf]

ChemistryOpen

Supporting Information

# Dynamic control of circumrotation of a [2]catenane by acid-base switching

Kelun Shi, Guohui Jia, Ying Wu, Shilong Zhang,* and Jiawen Chen*

Supporting Information

# Dynamic control of circumrotation of a [2]catenane by acid-base switching

Kelun Shi, Guohui Jia, Ying Wu, Shilong Zhang,* and Jiawen Chen*

# Table of Contents

1. General information ........ S1
2. Synthetic procedures of compounds ........ S1
3. Structural change of crown ether **3** by acid-base switching ........ S5
   3.1 NMR spectroscopy of crown ether **3** ........ S5
   3.2 The reversible switch between **3** and **3**-$H_2$·$2PF_6$ by acid-base ........ S13
   3.3 $^1$H NMR titration experiment of crown ether **3** ........ S14
4. Structural change of catenane by acid-base switching ........ S16
   4.1 NMR spectroscopy of catenane ........ S16
   4.2 The reversible switch between **1**-$H_2$·$6PF_6$ and **1**·$4PF_6$ by acid-base ........ S20
   4.3 $^1$H NMR titration experiment of catenane **1**-$H_2$·$6PF_6$ ........ S21
5. Determination of circumrotating rates of different states catenane ........ S23
   5.1 Determination of circumrotating rates of protonated catenane **1**-$H_2$·$6PF_6$ ........ S23
   5.2 Determination of circumrotating rates of deprotonated catenane **1**·$4PF_6$. ........ S25
6. UV-vis spectra of catenane by acid-base switching ........ S27
7. The $^1$H NMR and $^{13}$C NMR spectra of the compounds ........ S29
8. The MS spectra of the compounds ........ S35
9. Reference ........ S38

## 1. General information

**Instrument:** $^{1}$H NMR and $^{13}$C NMR data were recorded on a Bruker ADVANCE NEO 600 MHz testing at 298 K, and variable temperature NMR were recorded on a Bruker ADVANCE NEO 400 MHz. The chemical shifts (δ) for both $^{1}$H and $^{13}$C NMR are recorded in parts per million, with TMS serving as the internal standard. High resolution mass spectral data were obtained on Thermo Scientific Q Exactive. UV-vis absorption spectra were tested using a Lambda 950 spectrometer (PerkinElmer, Germany).

**Reagent and materials:** All reactions were achieved under an argon atmosphere unless otherwise indicated. All reagents were purchased from Energy Chemical or Bide Pharmatech and were used as received without further purification. Analytical thin-layer chromatography (TLC) was performed on Nuotai silica gel F254 plates and viewed under UV light.

## 2. Synthetic procedures of compounds

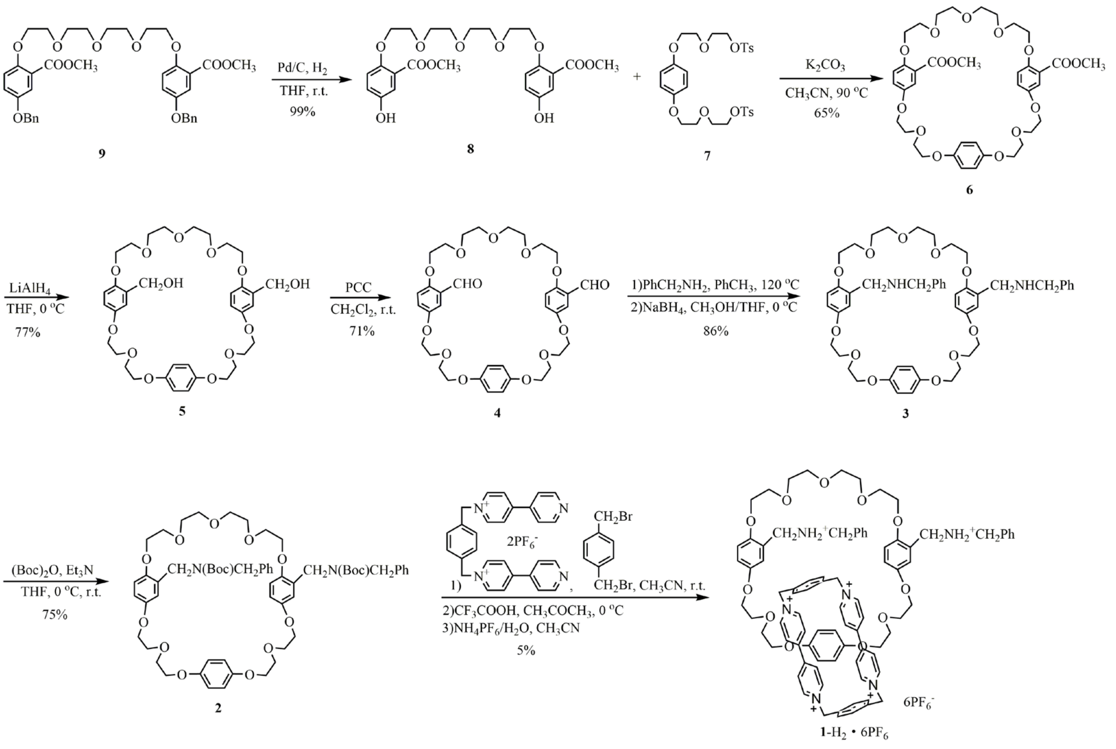


**Scheme S1.** The synthetic route of [2]catenane **1**-$H_2$ • $6PF_6$.

**Synthesis of 8**

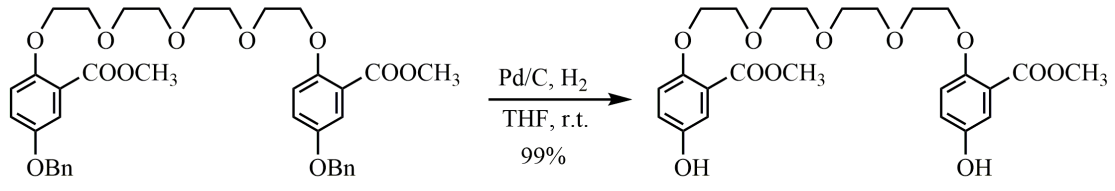


9 8

**9** (4 g, 5.93 mmol) was dissolved in THF (20 mL), added with 10% Pd/C (0.80 g), pressurized to 4 atmospheres under $H_2$ atmosphere at r.t., and reacted in Wattcas autoclave (WP-MSAR-250A) for 48 h. Then the filtrate was collected by vacuum filtration, and the solvent was evaporated to get compound **8** as a light yellow oil (2.90 g, yield: 99%), without further purification.

**Synthesis of 6**

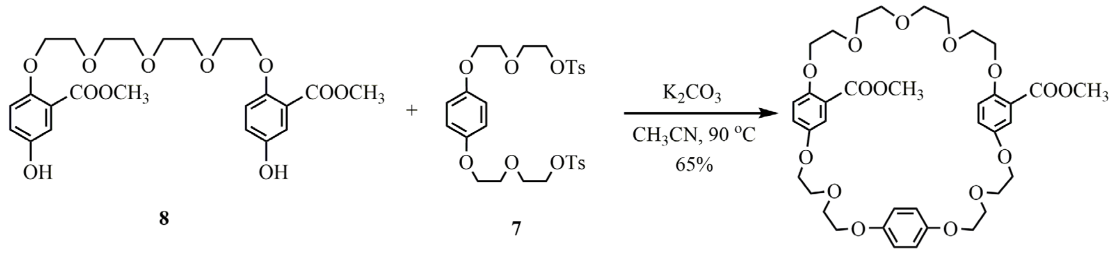


8 7 6

A suspension of **8** (0.83 g, 1.68 mmol), **7** (1.00 g, 1.68 mmol) and $K_2CO_3$ (1.16 g, 8.40 mmol) in dry $CH_3CN$ (100 mL), was stirred at 90 ℃ for 48 h. Then the filtrate was collected by vacuum filtration and the solvent was evaporated, which was separated by column chromatography [V(ethyl acetate):V(dichloromethane)=2:1], and the compound **6** was obtained as a light yellow solid (0.81 g, yield: 65%).

**Synthesis of 5**

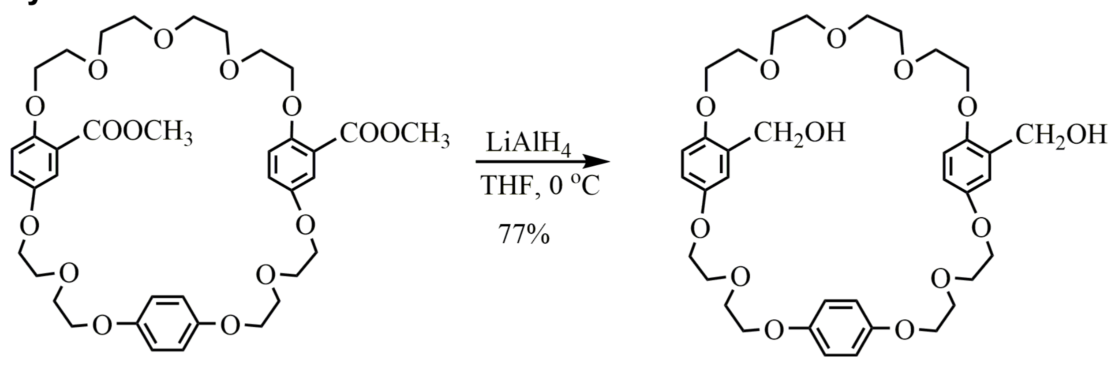


6 5

A solution was prepared by dissolving **6** (3.50 g, 4.70 mmol) in dry THF (15 mL), and the solution was slowly added to a flask containing $LiAlH_4$ (0.72 g, 18.80

mmol) at 0 ℃, and the reaction was maintained at 0 ℃ for more than 4 h. After the reaction was finished, ethyl acetate was slowly added at 0 ℃ until no bubbles were produced, and then 1 M HCl and $CH_2Cl_2$ were added at 0 ℃, stirring until there is no residue on the wall of the bottle, and the aqueous phase was extracted with $CH_2Cl_2$ for three times, and the organic phases were collected and dried with anhydrous $Na_2SO_4$, and then the solvent was evaporated and recrystallized with the solvent of $CH_3OH$ to get compound **5** as a light yellow solid (2.47 g, yield: 77%).

**Synthesis of 4**

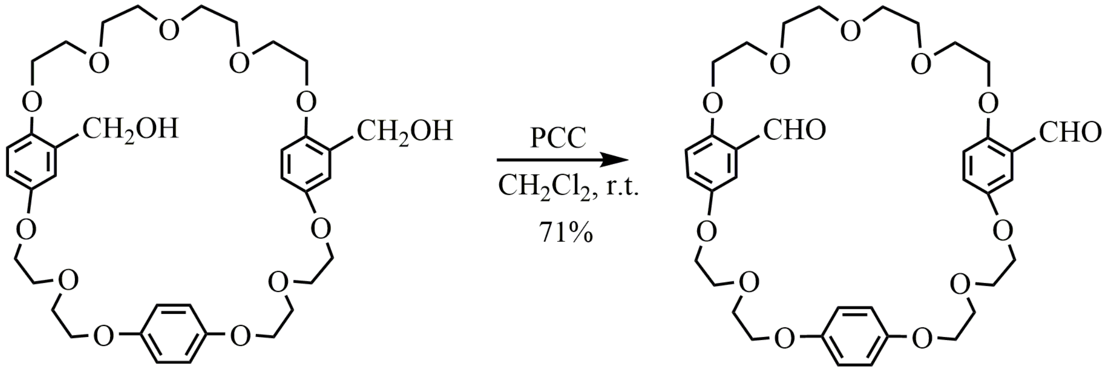


5 4

**5** (2.00 g, 1.45 mmol) and PCC (2.50 g, 5.81 mmol) were dissolved in $CH_2Cl_2$ (40 mL) and stirred at r.t. for more than 4 h. Then the filtrate was collected by vacuum filtration and the solvent was evaporated, which was separated by column chromatography [V(ethyl acetate):V(dichloromethane)=2:1] to obtain compound **4** as a white solid (1.41 g, yield: 71%).

**Synthesis of 3**

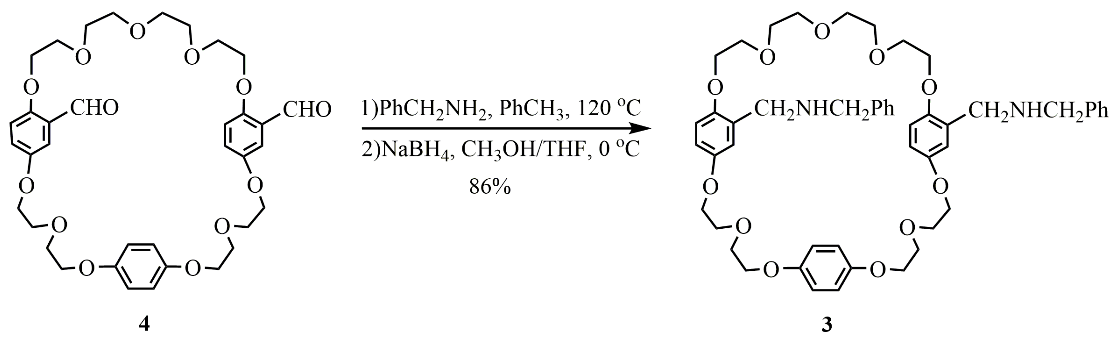


4 3

**4** (1.90 g, 2.78 mmol) and benzylamine (1.19 g, 11.10 mmol) were dissolved in dry $PhCH_3$ (20 mL) stirring at 120 ℃ for 12 h. After the reaction, the solvent was evaporated and the 5 mL $CH_3OH$ and 1 mL THF were added to dissolve rude product. Then $NaBH_4$ (0.63 g, 16.65 mmol) was added several times in a small amount at 0 ℃, and the reaction was maintained at 0 ℃ for more than 4 h. After the reaction was finished, distilled water and 1 M HCl were slowly added at 0 ℃ until no bubbles were produced, and the solvent was stirred for 30 min. Then

the aqueous phase was extracted with $CH_2Cl_2$ for 3 times, and the organic phases were collected and dried with anhydrous $Na_2SO_4$, and the solvent was removed to get compound **3** as a light yellow oil (2.07 g, yield: 86%).

**Synthesis of 2**

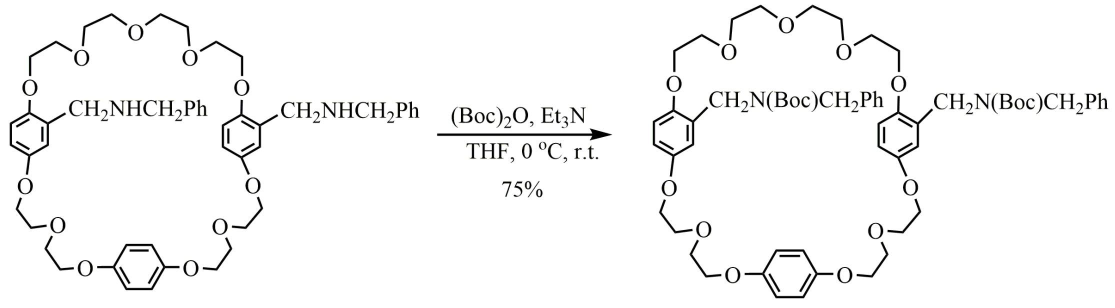


**3** (2.89 g, 3.33 mmol), $Et_3N$ (1.35 g, 13.32 mmol) and $(Boc)_2O$ (2.91 g, 13.32 mmol) were dissolved in dry THF (20 mL) stirring at 0 ℃ for 12 h. After the reaction, the solvent was evaporated and the $CH_2Cl_2$ was added to dissolve rude product, which was washed by distilled water twice, and then washed with saturated aqueous NaCl twice. The organic phase was collected and dried with anhydrous $Na_2SO_4$ and separated by column chromatography [V(ethyl acetate):V(dichloromethane)=1:4] to obtain compound **2** as a light yellow oil (2.67 g, yield: 75%).

**Synthesis of 1-H$_2$·6PF$_6$**

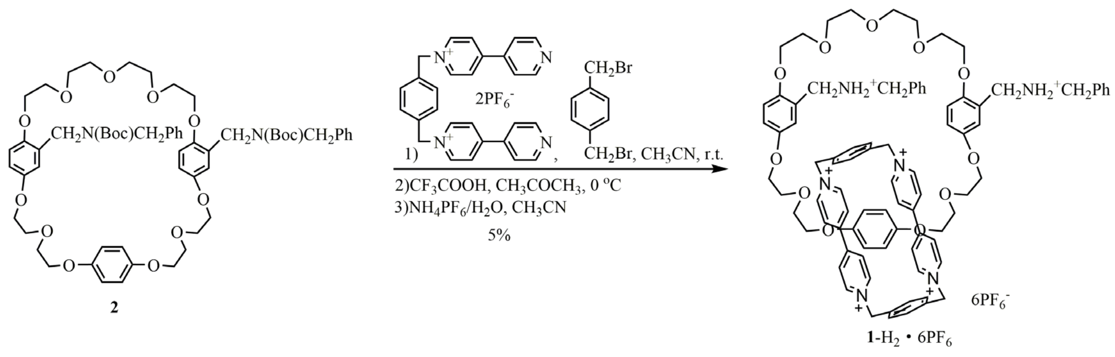


**2** (0.30 g, 0.28 mmol) was dissolved in dry $CH_3CN$ (6 mL) with p-xylylene-bis(4-(4-pyridyl)pyridinium) bis(hexafluorophosphate) (0.199 g, 0.28 mmol). After stirring for 1 h, 1,4-bis(bromomethyl)benzene (0.74 g, 0.28 mmol) was added and stirred at r.t. for 15 d. After the reaction, the red solid was obtained by column chromatography [V(MeOH):V(2 M $NH_4Cl$):V($CH_3NO_2$)=7:2:1]. The solid was dissolved in $CH_3COCH_3$, and $CF_3COOH$ (0.22 mL, 2.80 mmol) was added and stirred at 0 ℃ for more than 4 h. Then the solvent was evaporated, and the crude product was dissolved with a small amount of $CH_3CN$, and saturated aqueous $NH_4PF_6$ was added until no solid was precipitated. By vacuum filtration, the solid was collected, then recrystallized with $CH_3CN/H_2O$ to obtain a red solid (31.64 mg, yield: 5%).

# 3. Structural change of crown ether 3 by acid-base switching

## 3.1 NMR spectroscopy of crown ether 3

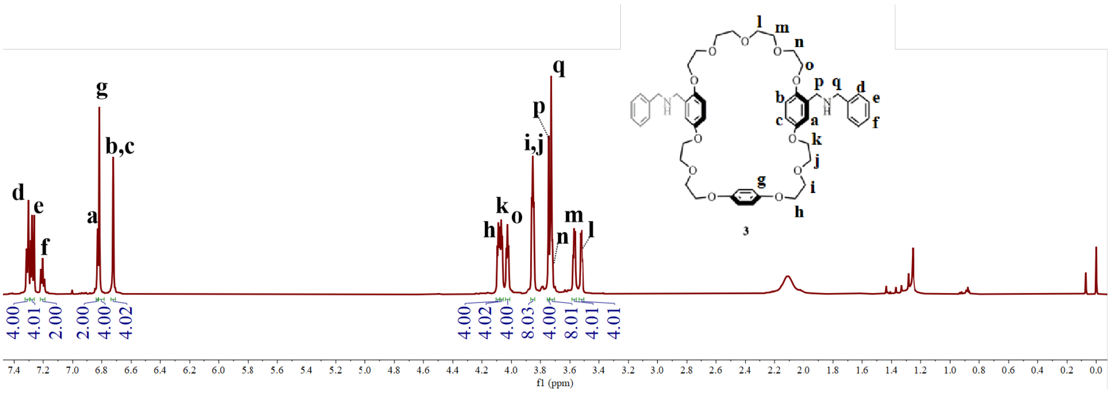


**Figure S1.** $^1$H NMR spectrum (600 MHz, $CDCl_3$, 298 K) of compound **3**.

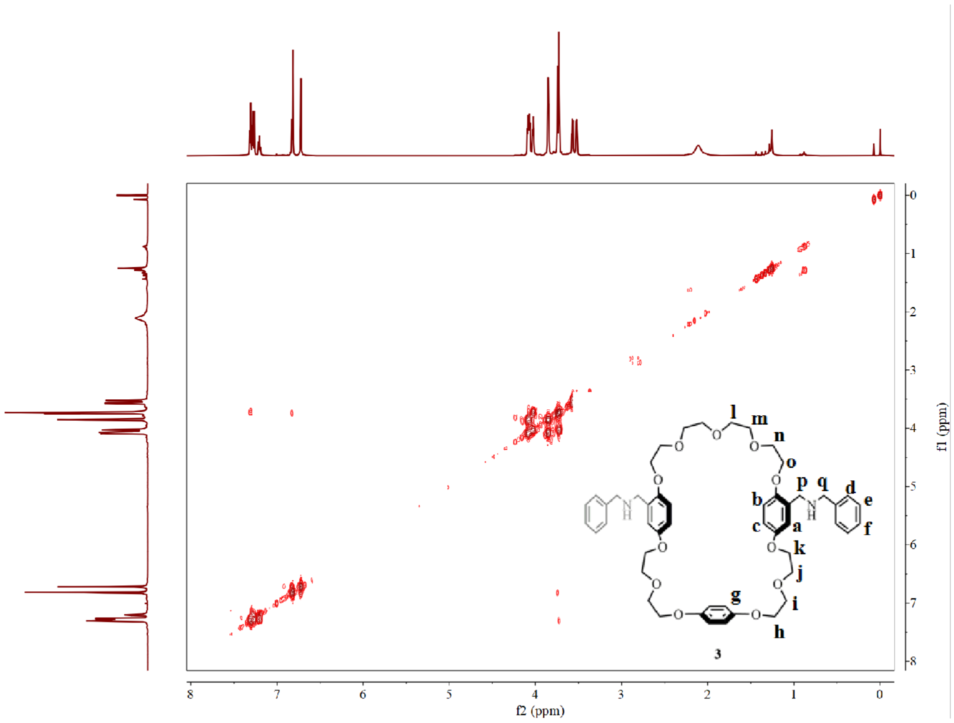


**Figure S2.** $^1$H-$^1$H COSY spectrum (600 MHz, $CDCl_3$, 298 K) of compound **3**.

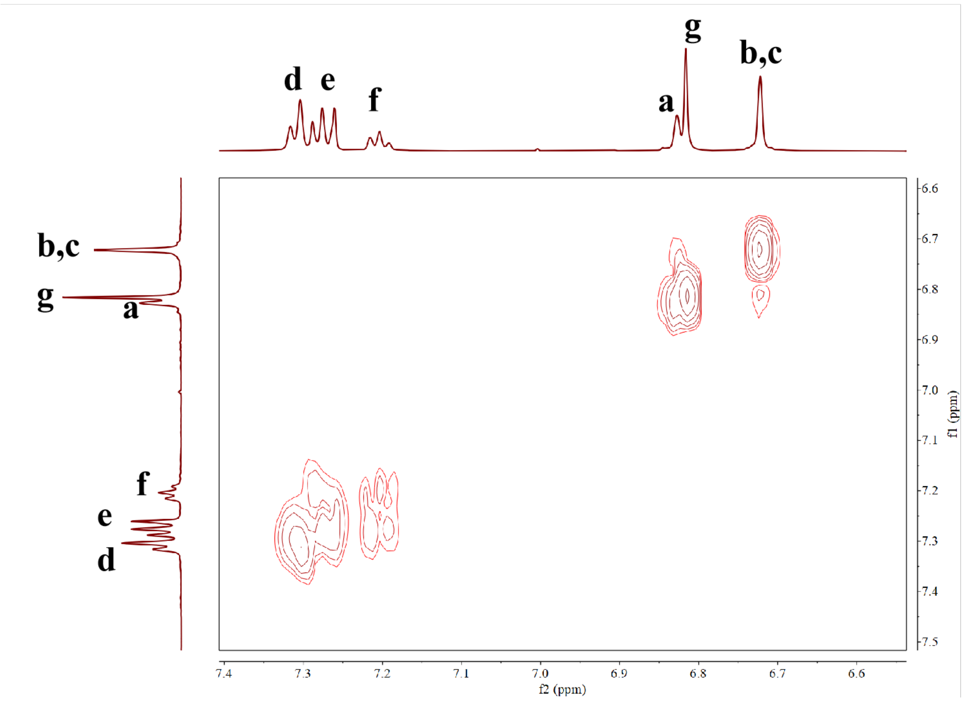


**Figure S3.** Partial $^{1}$H-$^{1}$H COSY spectrum (600 MHz, $CDCl_3$, 298 K) of compound **3**.

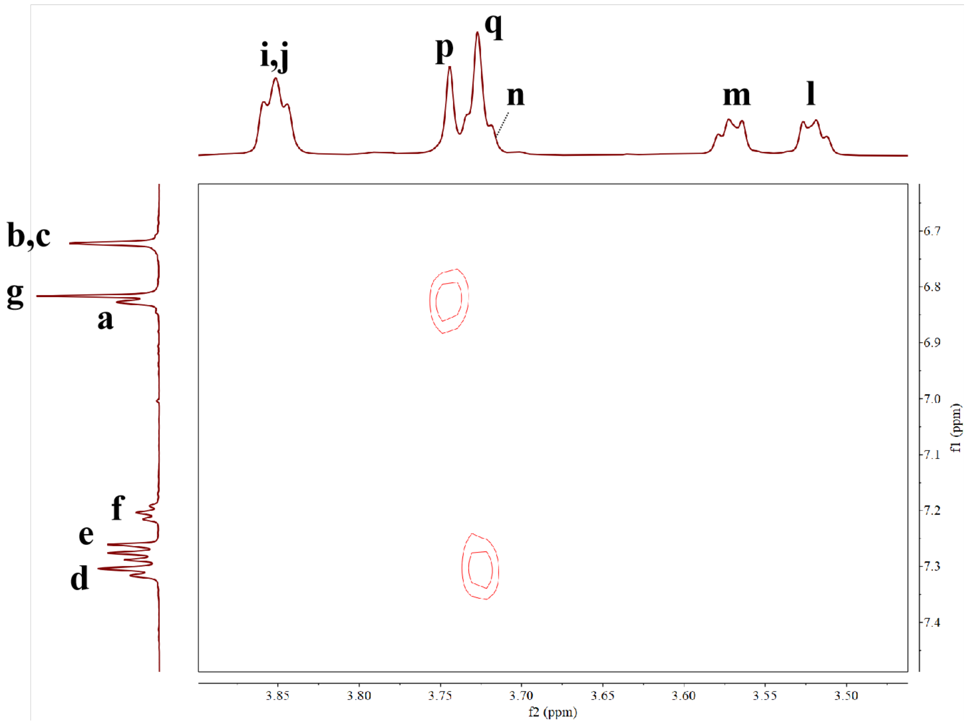


**Figure S4.** Partial $^{1}$H-$^{1}$H COSY spectrum (600 MHz, $CDCl_3$, 298 K) of compound **3**.

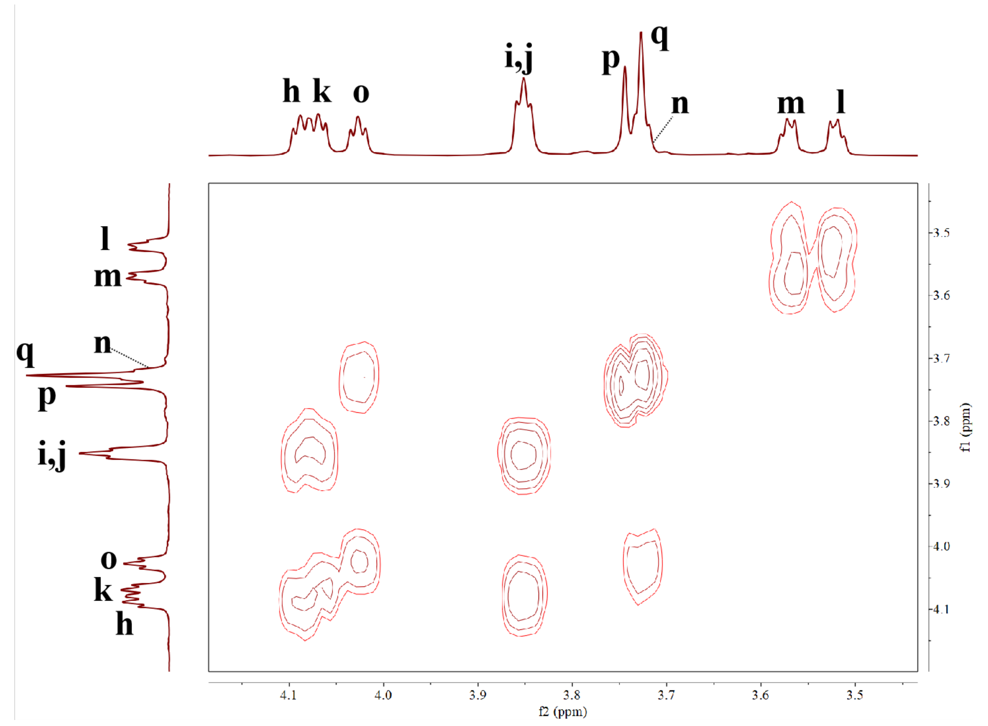


**Figure S5.** Partial $^1H$-$^1H$ COSY spectrum (600 MHz, $CDCl_3$, 298 K) of compound **3**.

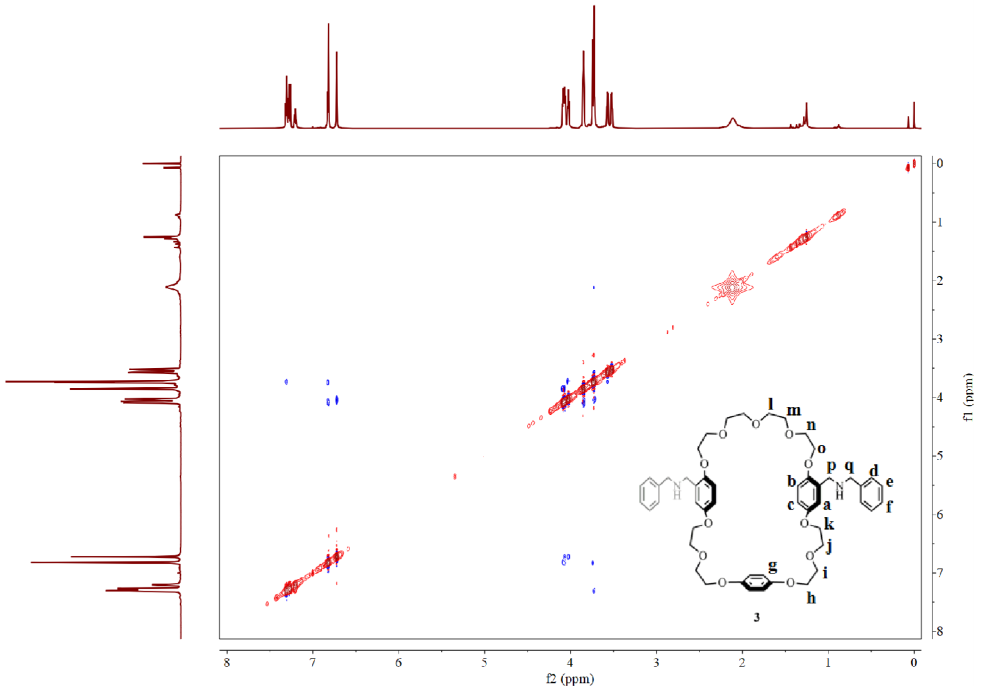


**Figure S6.** $^1H$-$^1H$ NOESY spectrum (600 MHz, $CDCl_3$, 298 K) of compound **3**.

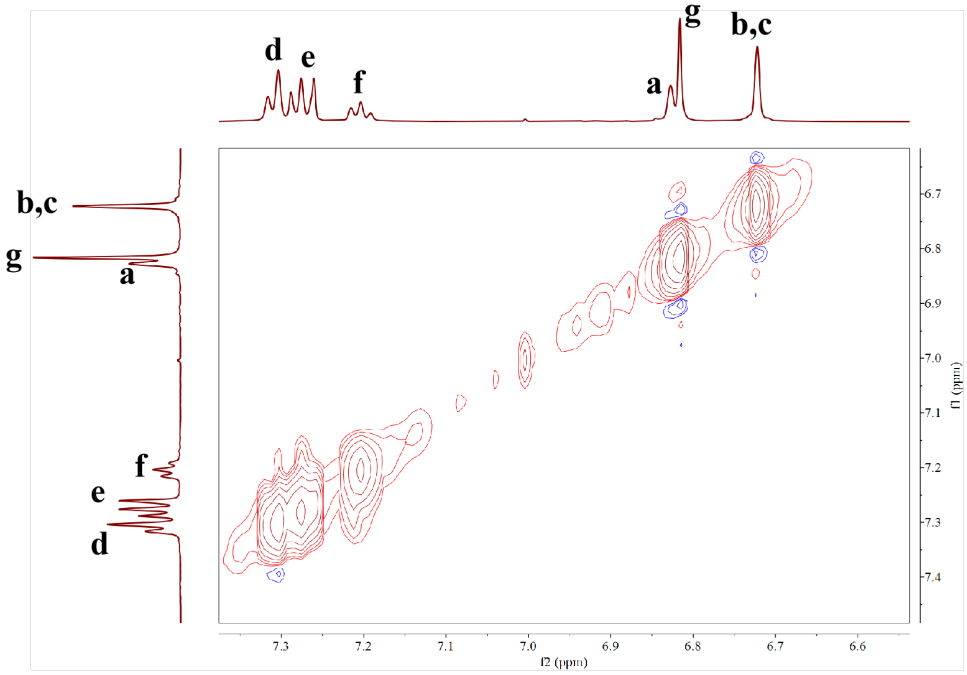


**Figure S7.** Partial $^1H$-$^1H$ NOESY spectrum (600 MHz, $CDCl_3$, 298 K) of compound **3**.

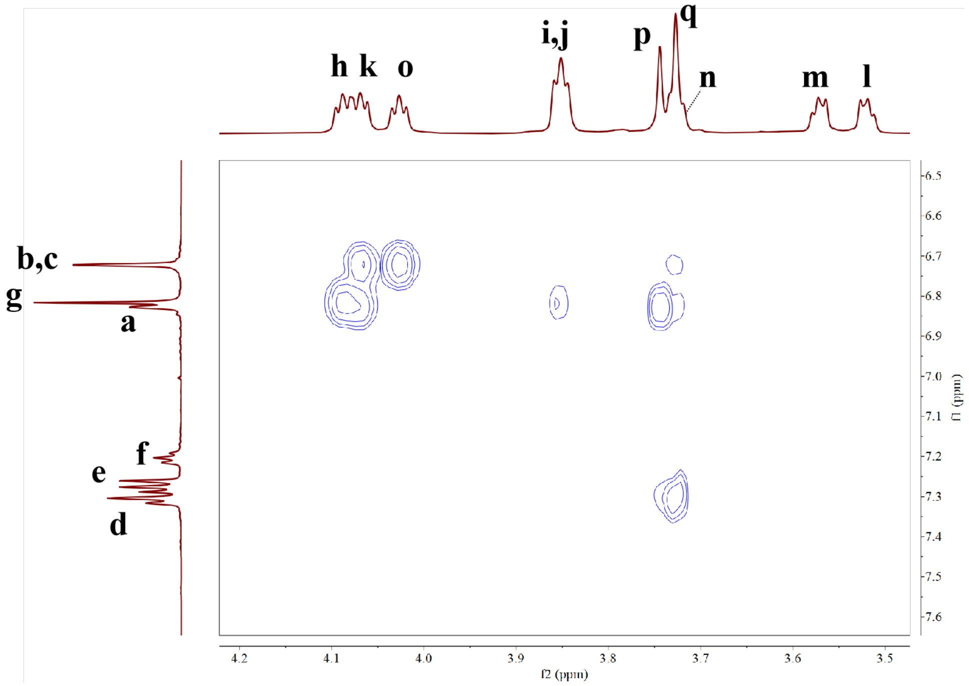


**Figure S8.** Partial $^1H$-$^1H$ NOESY spectrum (600 MHz, $CDCl_3$, 298 K) of compound **3**.

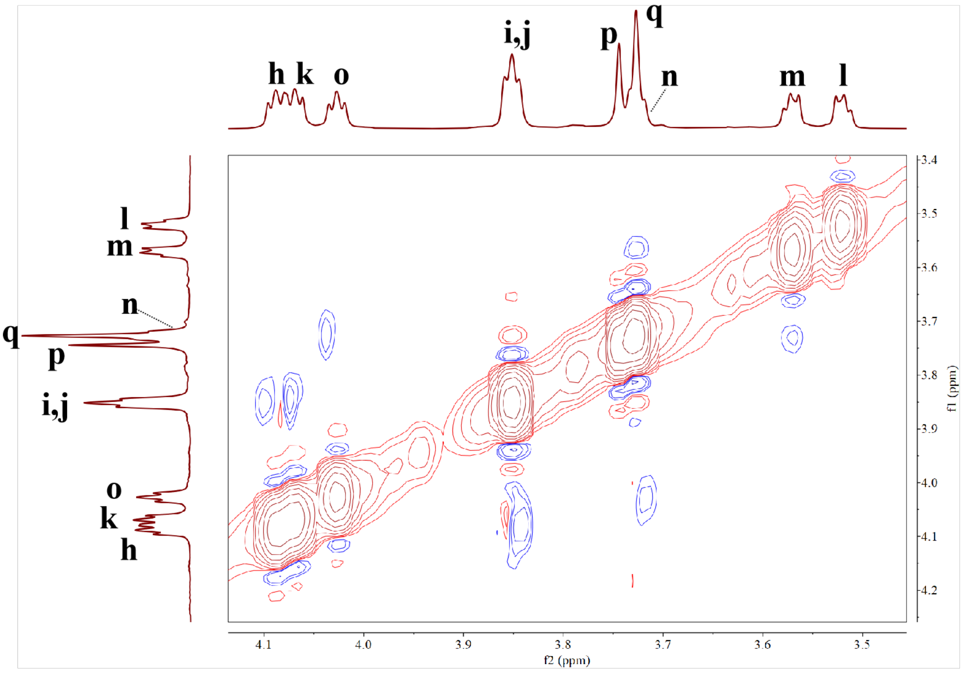


**Figure S9.** Partial $^{1}H$-$^{1}H$ NOESY spectrum (600 MHz, $CDCl_3$, 298 K) of compound **3**.

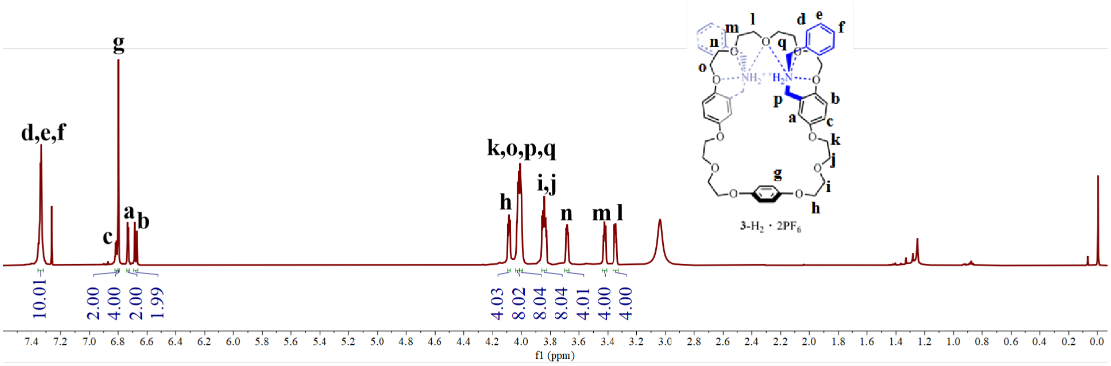


**Figure S10.** $^{1}H$ NMR spectrum (600 MHz, $CDCl_3$, 298 K) of **3**-$H_2$·$2PF_6$.

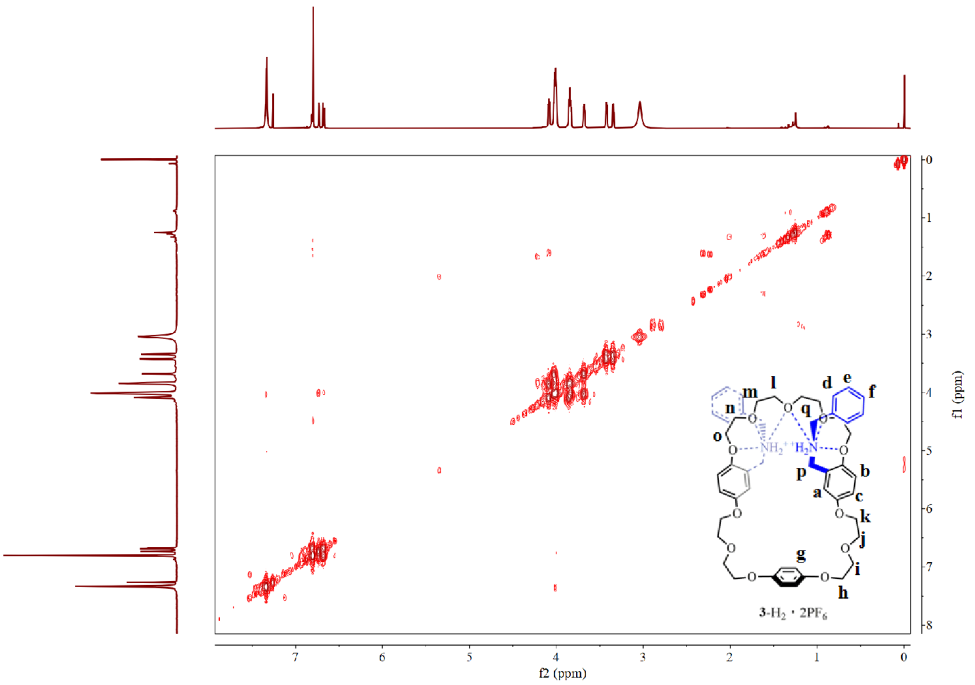


**Figure S11.** $^1$H-$^1$H COSY spectrum (600 MHz, $CDCl_3$, 298 K) of **3**-$H_2$·2$PF_6$.

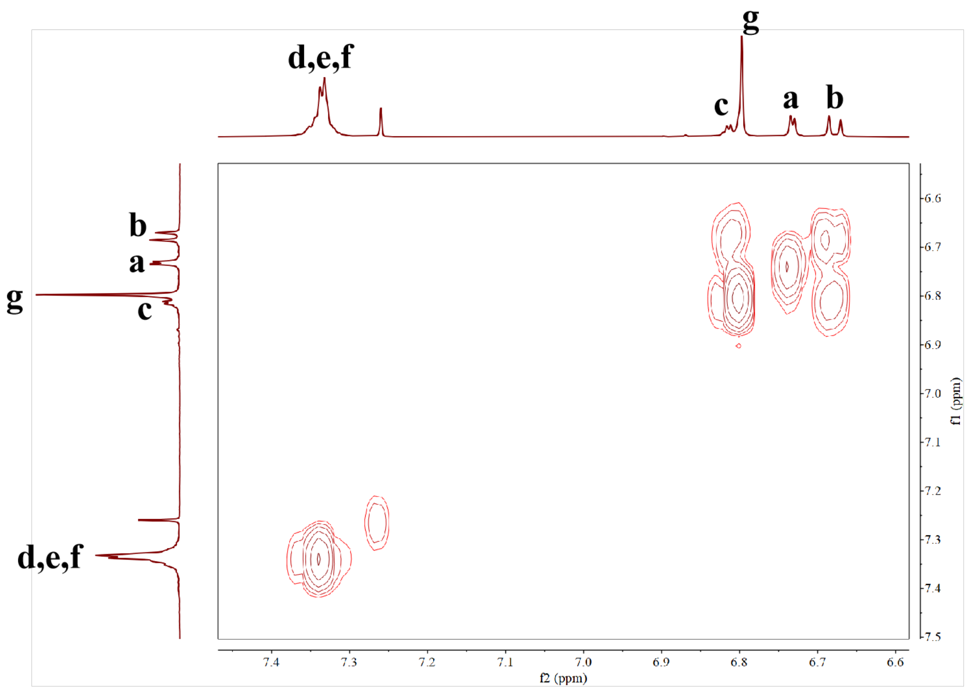


**Figure S12.** Partial $^1$H-$^1$H COSY spectrum (600 MHz, $CDCl_3$, 298 K) of **3**-$H_2$·2$PF_6$.

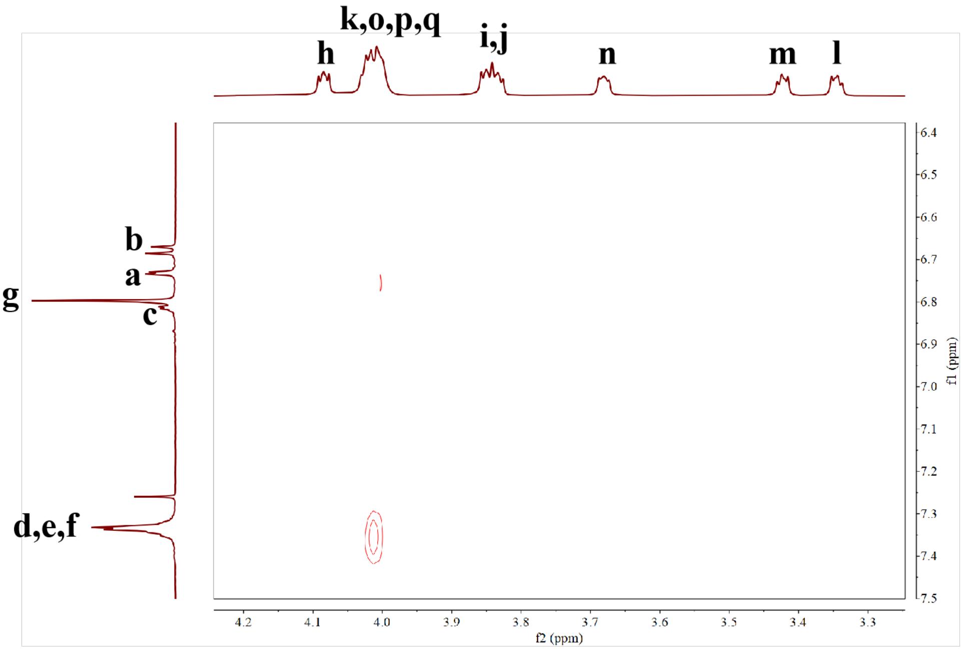


**Figure S13.** Partial $^1H$-$^1H$ COSY spectrum (600 MHz, $CDCl_3$, 298 K) of **3**-$H_2{\cdot}2PF_6$.

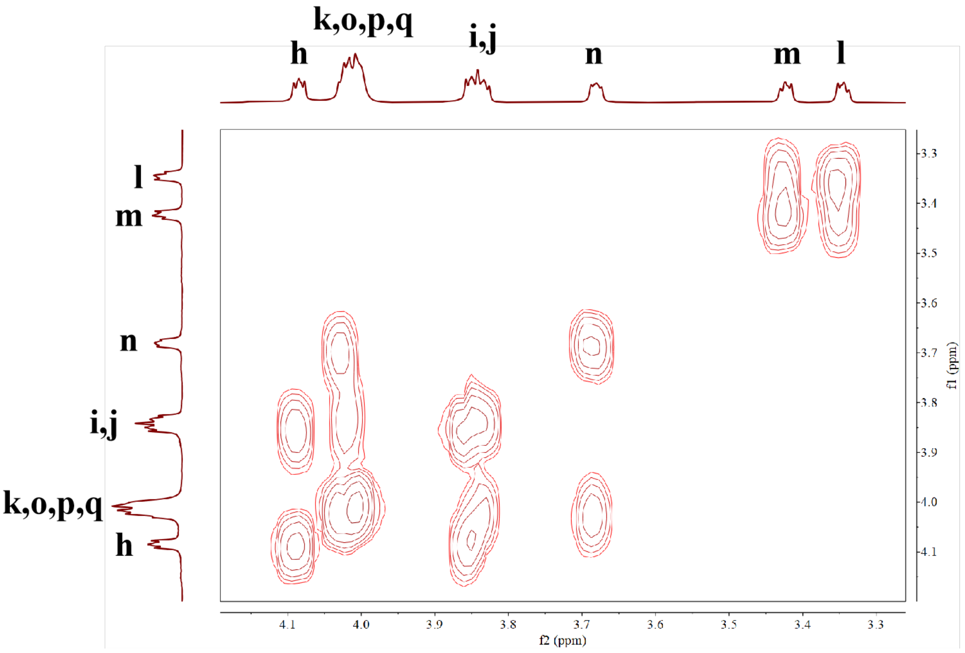


**Figure S14**. Partial $^1H$-$^1H$ COSY spectrum (600 MHz, $CDCl_3$, 298 K) of **3**-$H_2{\cdot}2PF_6$.

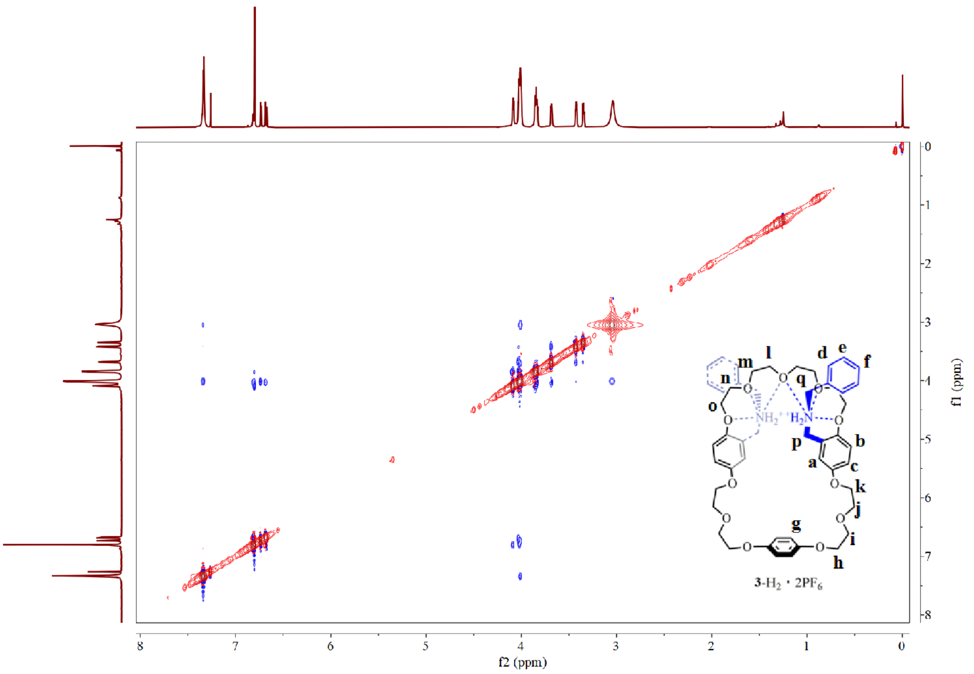


**Figure S15.** $^1$H-$^1$H NOESY spectrum (600 MHz, $CDCl_3$, 298 K) of **3**-$H_2$·$2PF_6$.

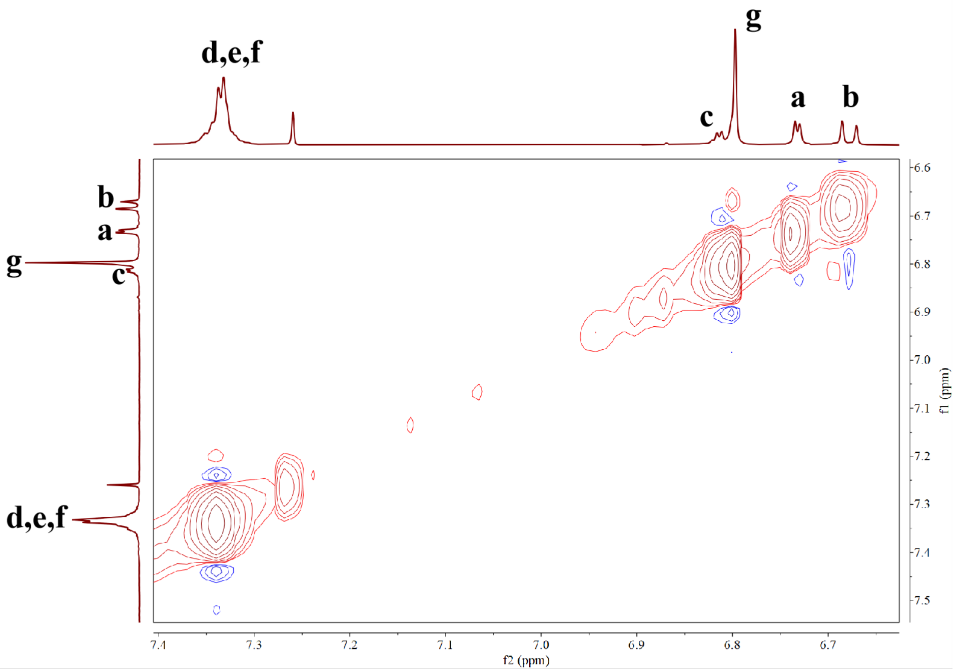


**Figure S16.** Partial $^1$H-$^1$H NOESY spectrum (600 MHz, $CDCl_3$, 298 K) of **3**-$H_2$·$2PF_6$.

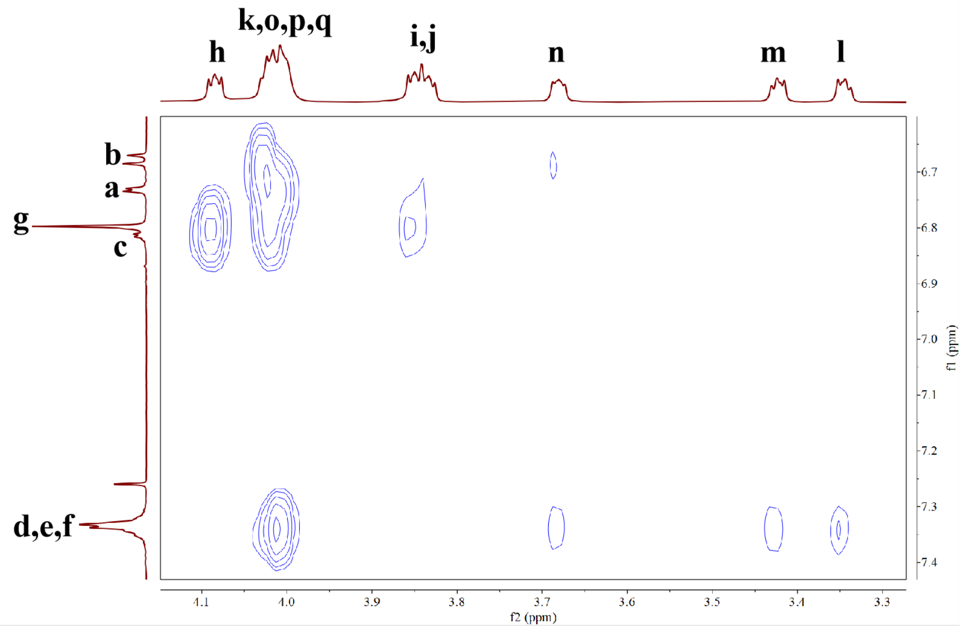


**Figure S17.** Partial $^{1}H$-$^{1}H$ NOESY spectrum (600 MHz, $CDCl_3$, 298 K) of **3**-$H_2$·$2PF_6$.

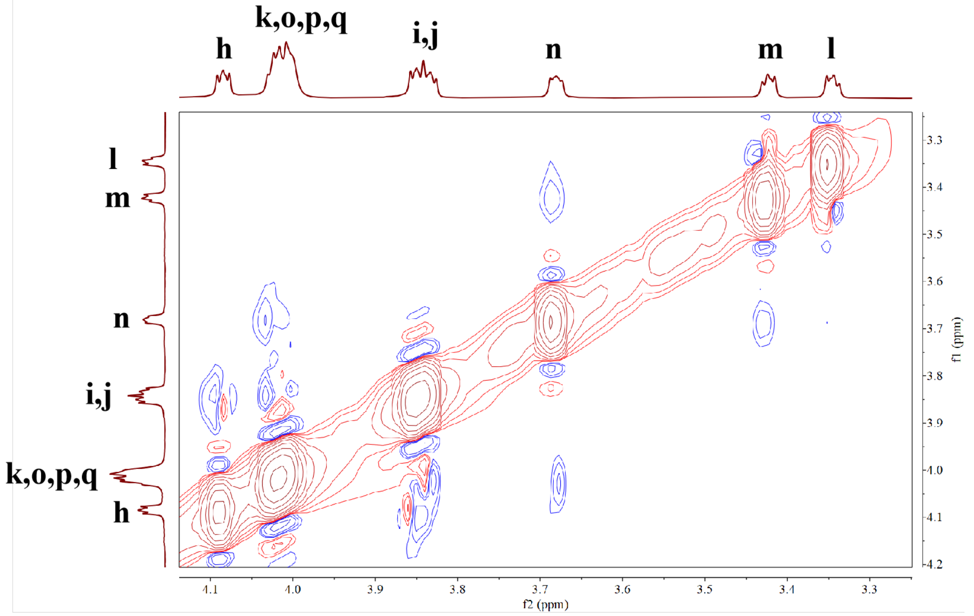


**Figure S18.** Partial $^{1}H$-$^{1}H$ NOESY spectrum (600 MHz, $CDCl_3$, 298 K) of **3**-$H_2$·$2PF_6$.

## 3.2 The reversible switch between 3 and 3-$H_2$·$2PF_6$ by acid-base

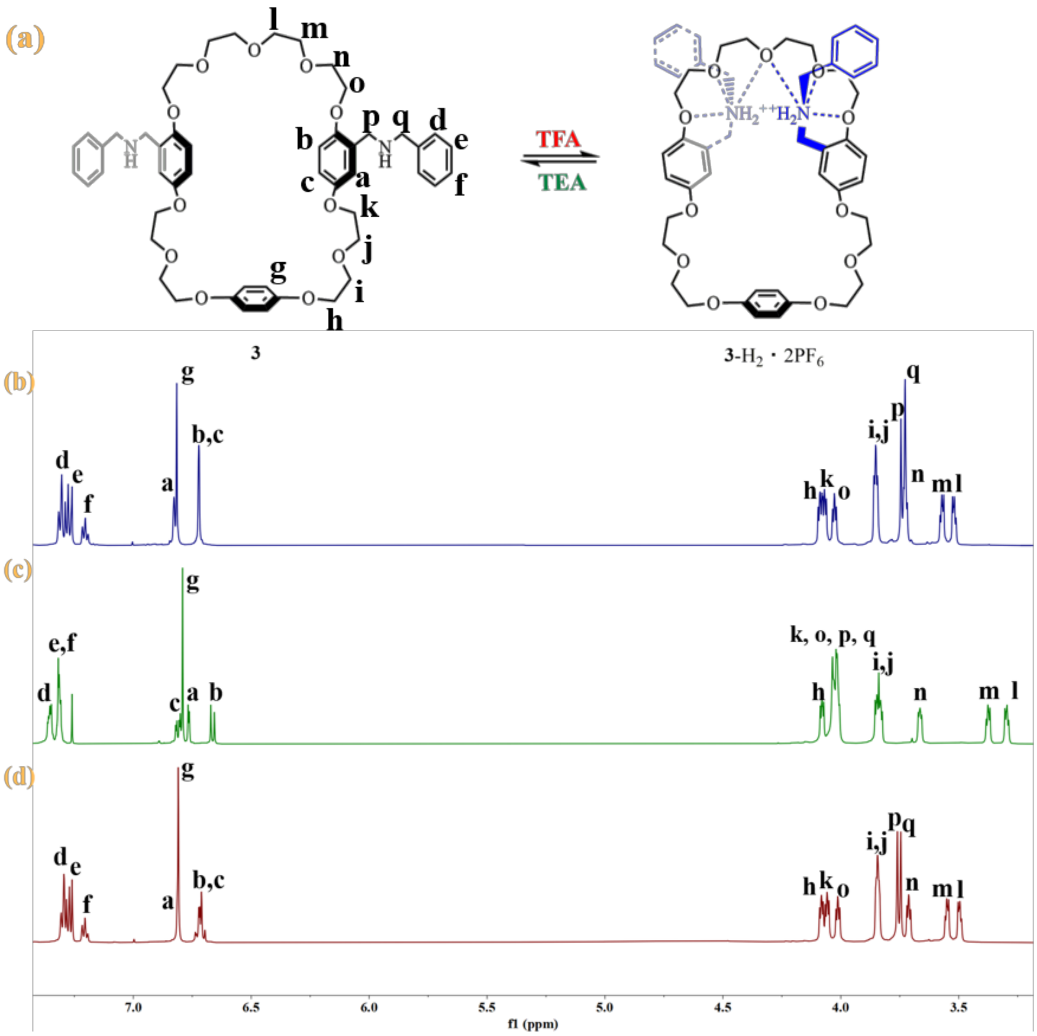


**Figure S19.** (a) Schematic representation the change of the cavity of crown ether **3** by acid-base switching, (b) $^1H$ NMR spectra (600 MHz, 298 K, $CDCl_3$) of 11.53 $\times 10^{-6}$ M compound **3**, (c) the solution obtained after addition of 3.0 equiv. of TFA to part (b), (d) the solution obtained after addition of 4.0 equiv. of TEA to part (c).

### 3.3 $^1H$ NMR titration experiment of crown ether 3

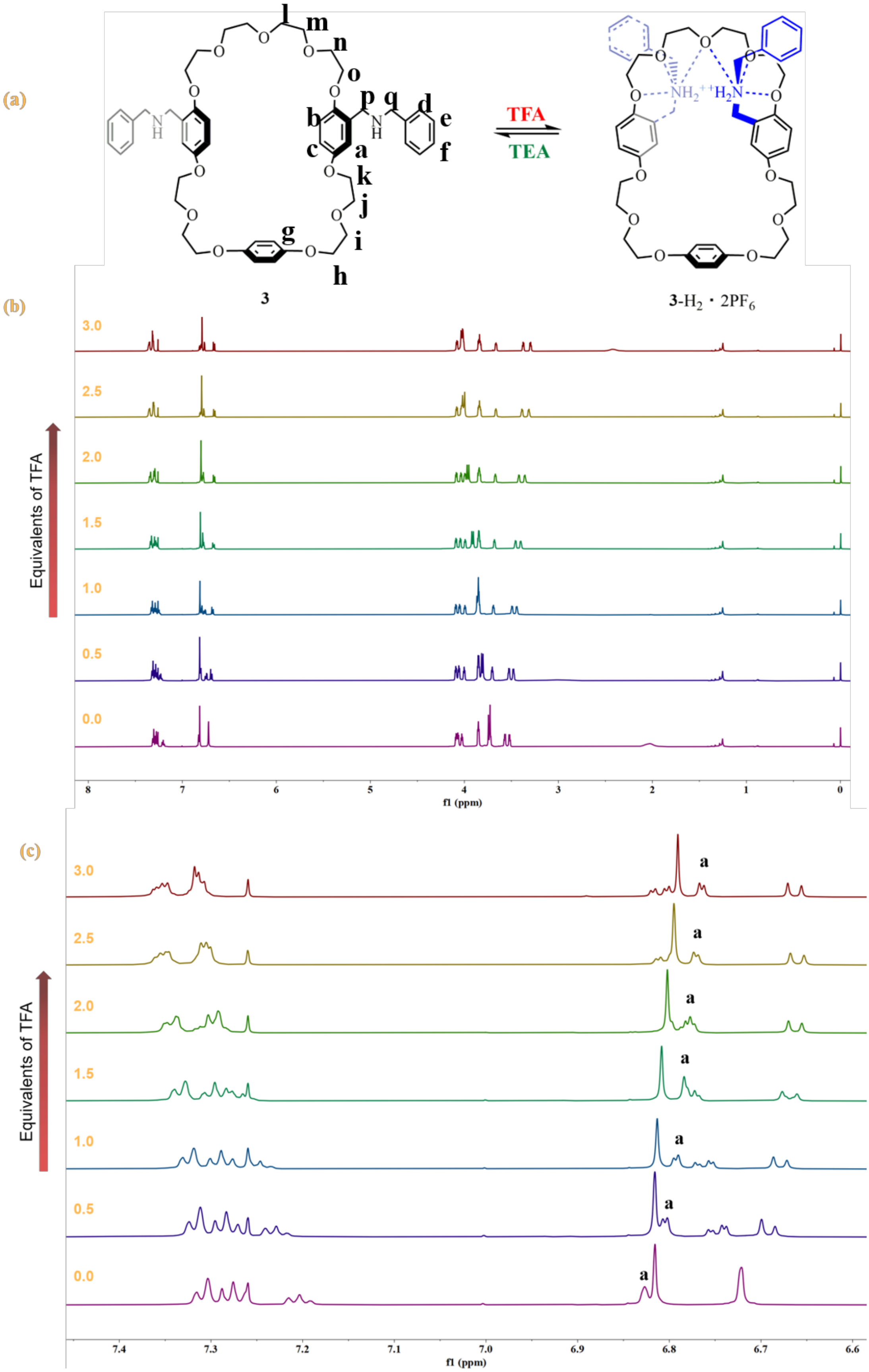


**Figure S20.** (a) Schematic representation the structures of different states of crown ether **3** by acid-base switching, (b) $^{1}H$ NMR spectra (600 MHz, 298 K, $CDCl_3$) of 11.53 x$10^{-6}$ M

compound **3** during the titration by TFA, (c) $^{1}$H NMR signals of proton $H_a$ during the titration by TFA.

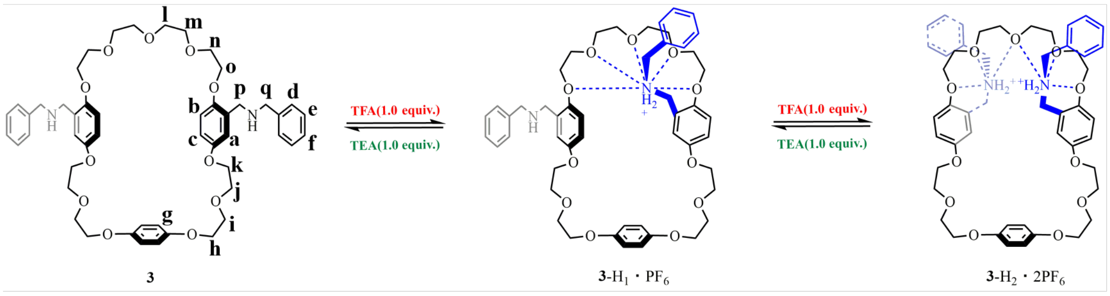


**Scheme S2**. The structures of different states of crown ether **3** by acid-base switching.

# 4. Structural change of catenane by acid-base switching

## 4.1 NMR spectroscopy of catenane

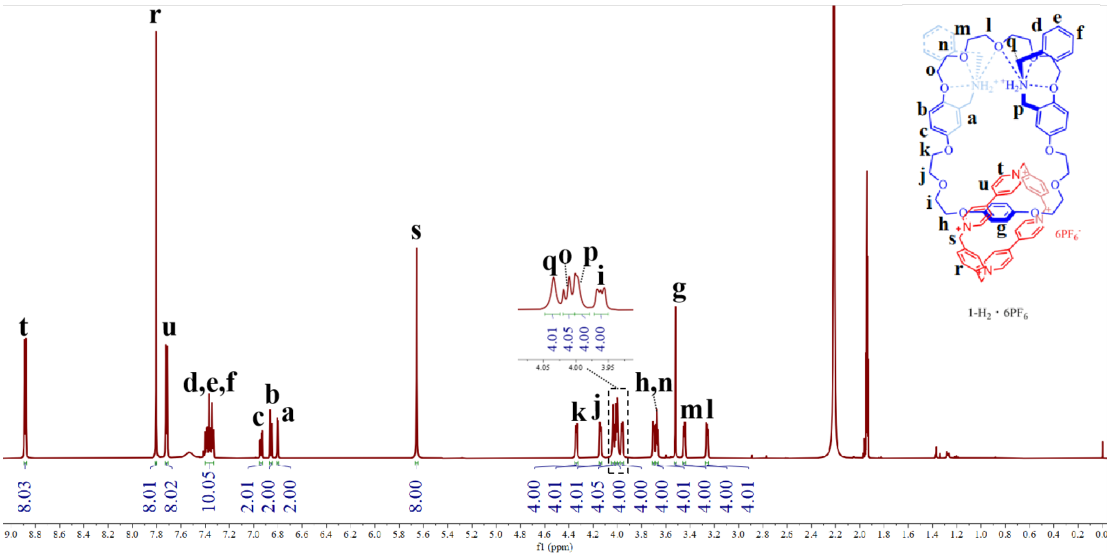


**Figure S21.** $^{1}$H NMR spectrum (600 MHz, $CD_3CN$, 298 K) of [2]catenane **1**-$H_2$·$6PF_6$.

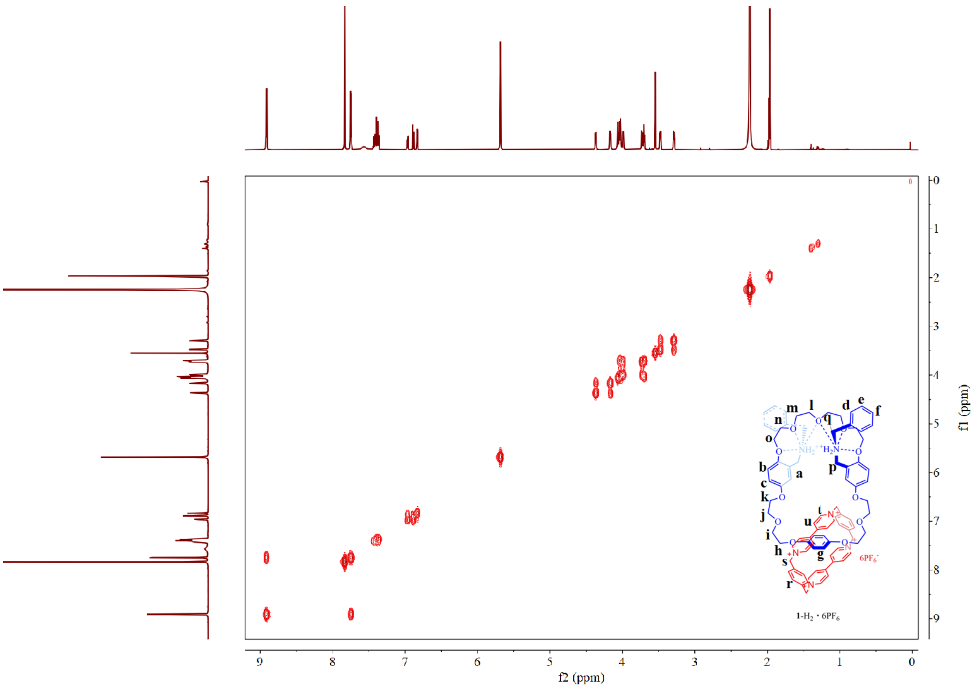


**Figure S22.** ¹H-¹H COSY spectrum (600 MHz, $CD_3CN$, 298 K) of [2]catenane **1**-$H_2{\cdot}6PF_6$.

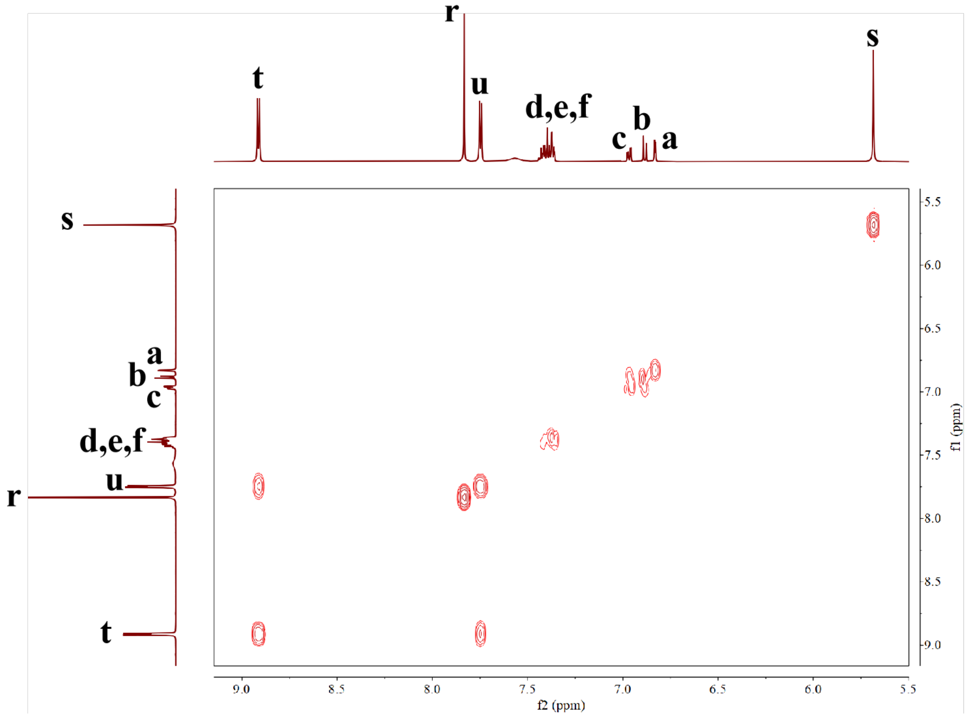


**Figure S23.** Partial ¹H-¹H COSY spectrum (600 MHz, $CD_3CN$, 298 K) of [2]catenane **1**-$H_2{\cdot}6PF_6$.

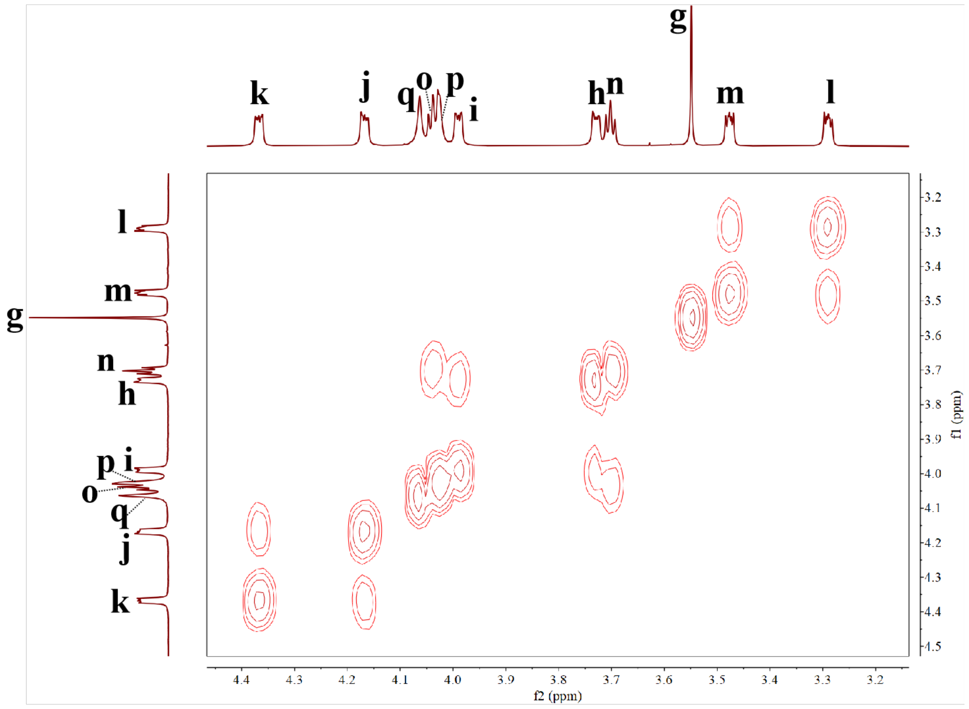


**Figure S24.** Partial $^1H$-$^1H$ COSY spectrum (600 MHz, $CD_3CN$, 298 K) of [2]catenane **1**-$H_2$·$6PF_6$.

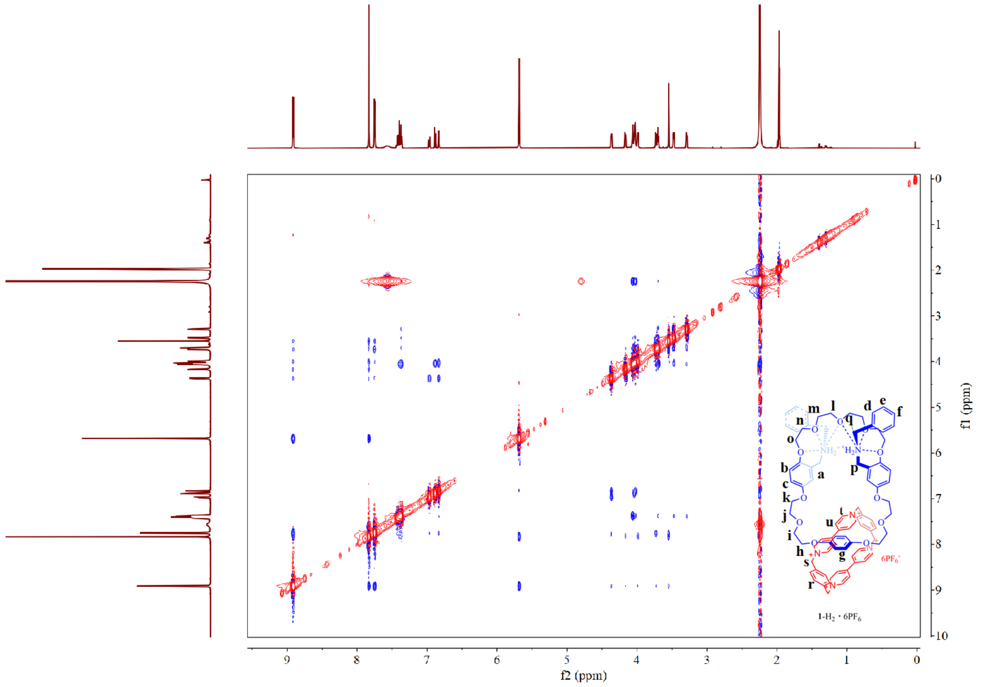


**Figure S25.** $^1H$-$^1H$ NOESY spectrum (600 MHz, $CD_3CN$, 298 K) of [2]catenane **1**-$H_2$·$6PF_6$.

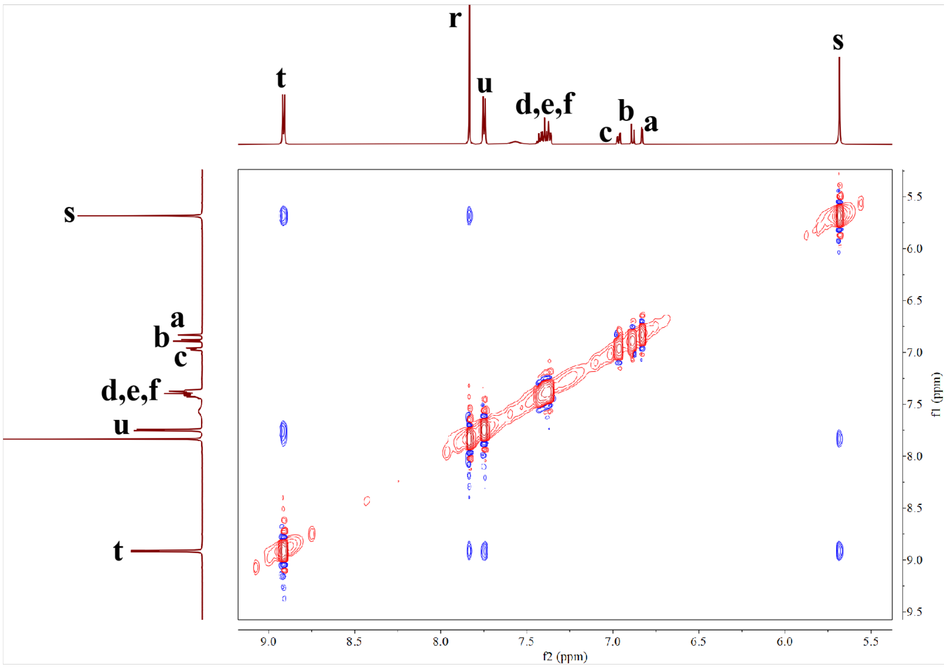


**Figure S26.** Partial $^1H$-$^1H$ NOESY spectrum (600 MHz, $CD_3CN$, 298 K) of [2]catenane **1**-$H_2{\cdot}6PF_6$.

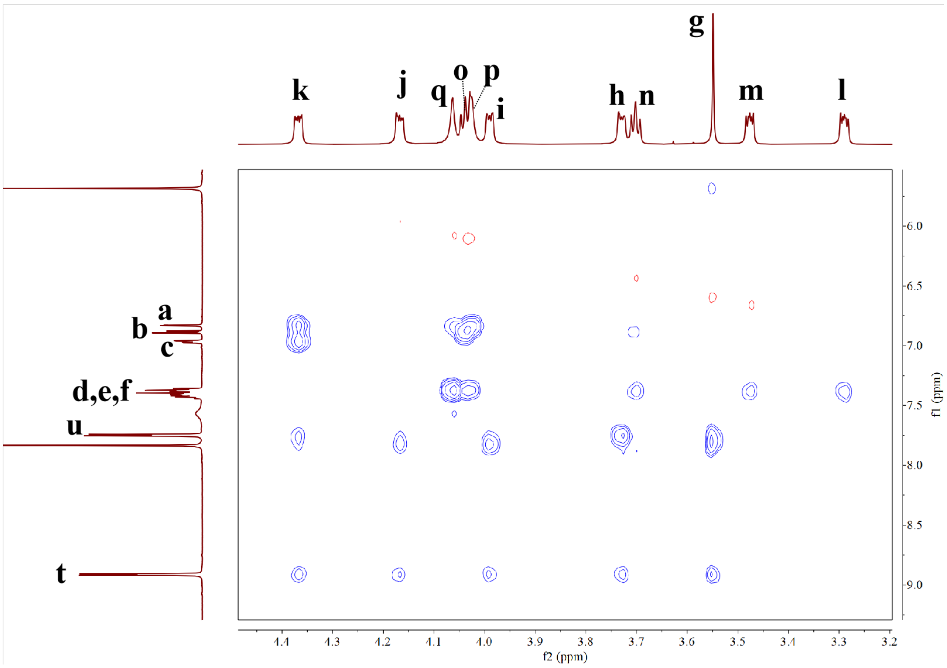


**Figure S27.** Partial $^1H$-$^1H$ NOESY spectrum (600 MHz, $CD_3CN$, 298 K) of [2]catenane **1**-$H_2{\cdot}6PF_6$.

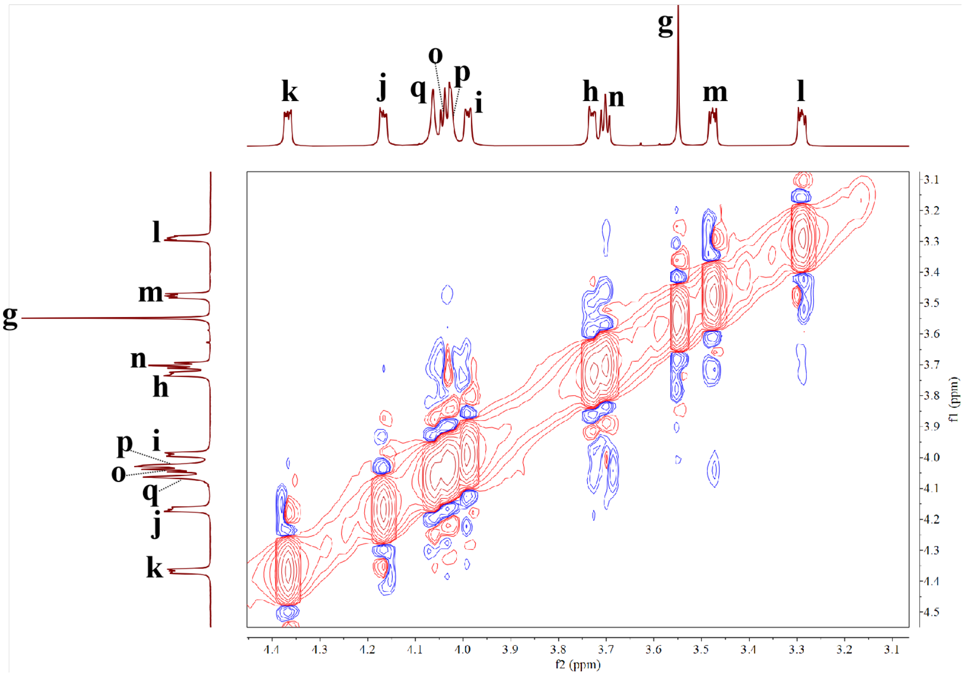


**Figure S28.** Partial $^{1}H$-$^{1}H$ NOESY spectrum (600 MHz, $CD_3CN$, 298 K) of [2]catenane **1**-$H_2 \cdot 6PF_6$.

## 4.2 The reversible switch between 1-$H_2 \cdot 6PF_6$ and 1·$4PF_6$ by acid-base

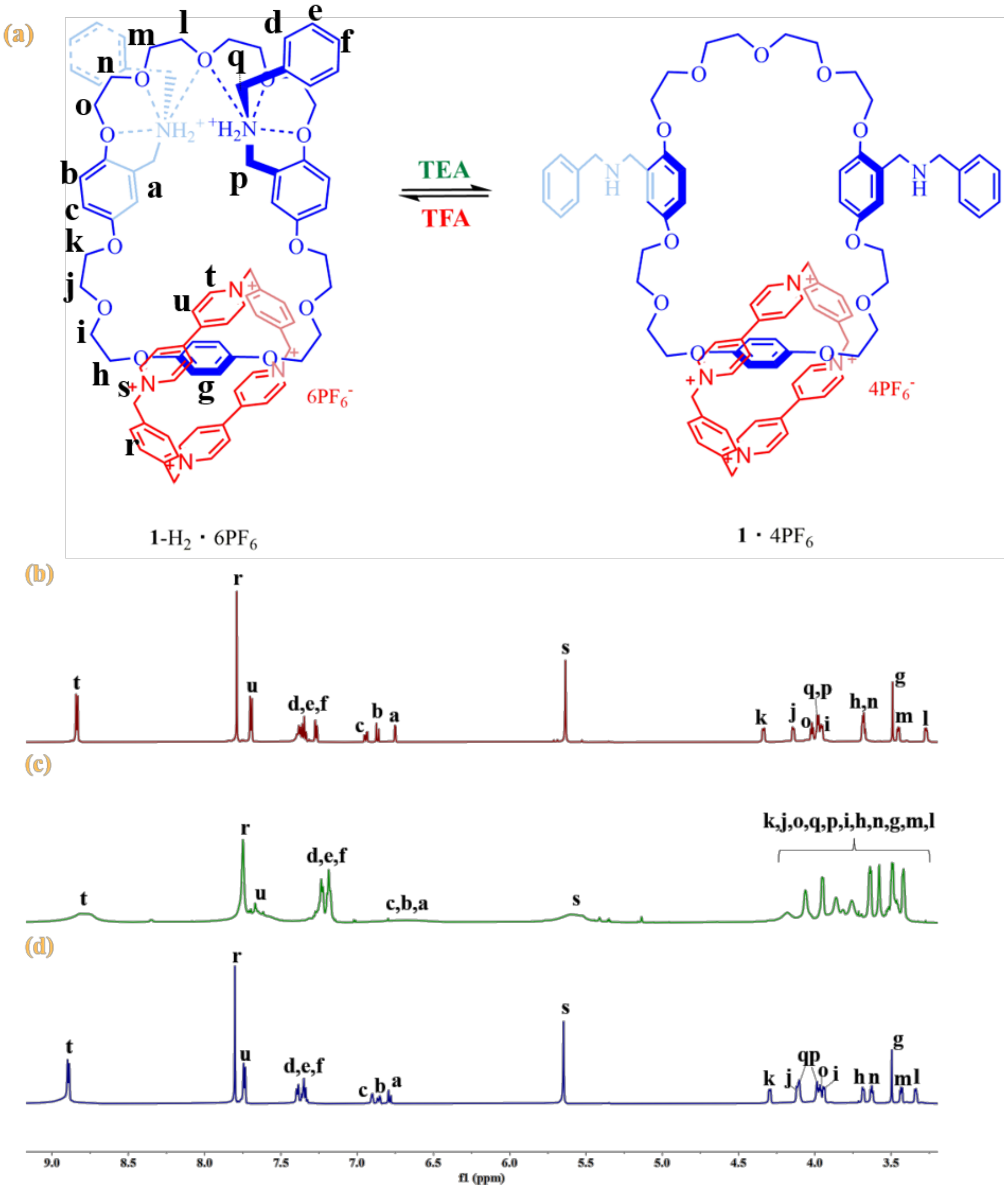


**Figure S29.** (a) Scheme representation the change of the cavity of catenane by acid-base switching, (b) $^{1}$H NMR spectra (600 MHz, 298 K, $CD_3CN$) of 7.52 x$10^{-6}$ M **1**-$H_2$·$6PF_6$, (c) the solution obtained after addition of 3.0 equiv. of TEA to part (b), (d) the solution obtained after addition of 6.0 equiv. of TFA to part (c).

## 4.3 $^{1}$H NMR titration experiment of catenane 1-$H_2$·$6PF_6$

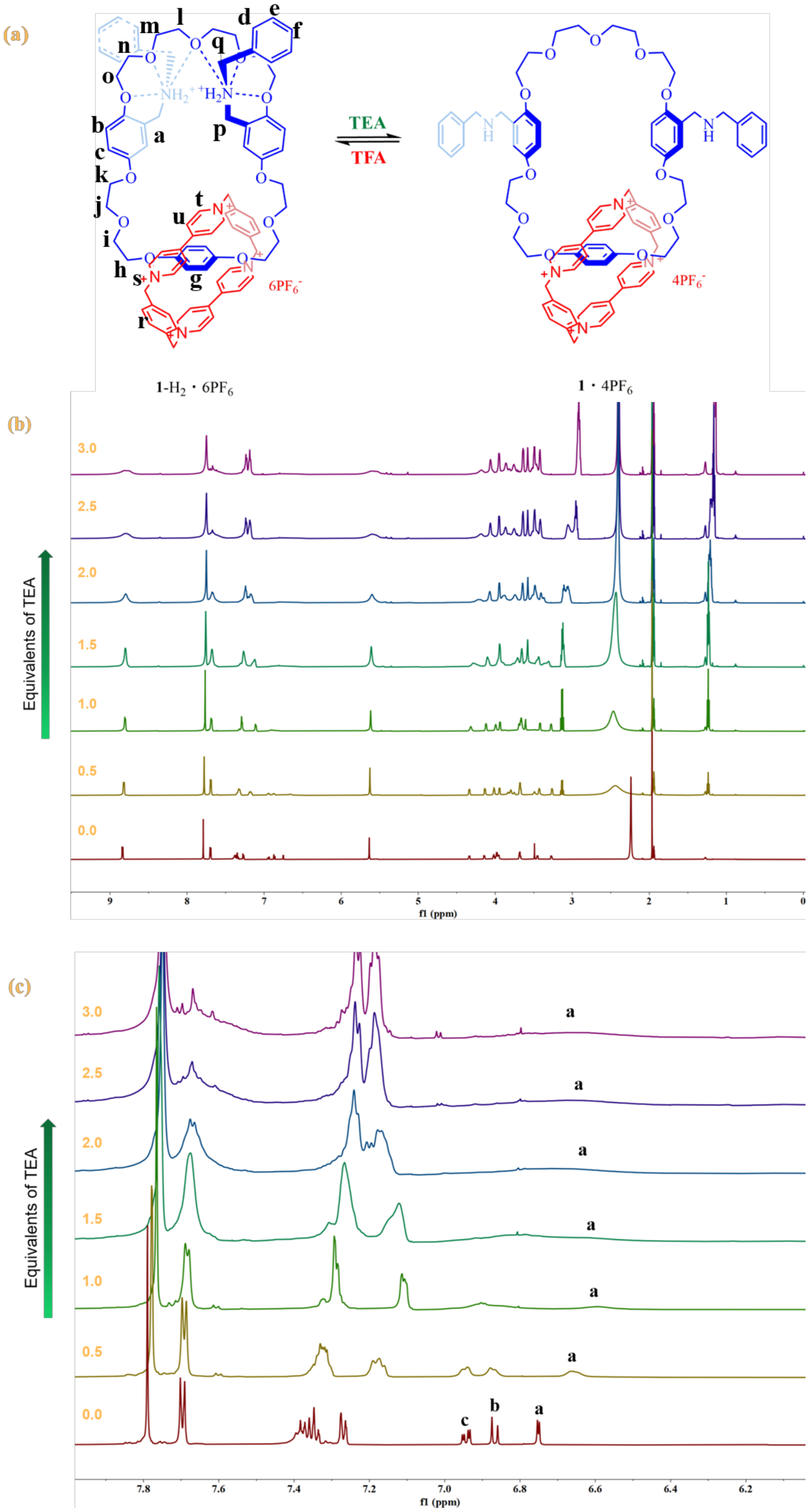


**Figure S30.** (a) Schematic representation the structures of different states of catenane by acid-base switching, (b) $^{1}$H NMR spectra (600 MHz, 298 K, $CD_3CN$) of 7.52 x$10^{-6}$ M **1-**

$H_2$·6$PF_6$ during the titration by TEA, (c) $^1H$ NMR signals of proton $H_a$ during the titration by TEA.

# 5. Determination of circumrotating rates of different states catenane

Variable temperature NMR experiment was applied to determine the circumrotation rate of the catenane. $^1H$ NMR spectra were recorded on a Bruker ADVANCE NEO 400 MHz within a temperature range from 203 K to 314.5 K. Proton t was selected to be used as the probe. Lineshape analysis was carried with the program D-NMR. Simulations were performed with exchange signals of proton t assuming a Gaussian lineshape. The values of $\Delta G^{\neq}$, $\Delta H^{\neq}$ and $\Delta S^{\neq}$ were determined by Eyring equation.[1-2]

## 5.1 Determination of circumrotating rates of protonated catenane 1-$H_2$·6$PF_6$

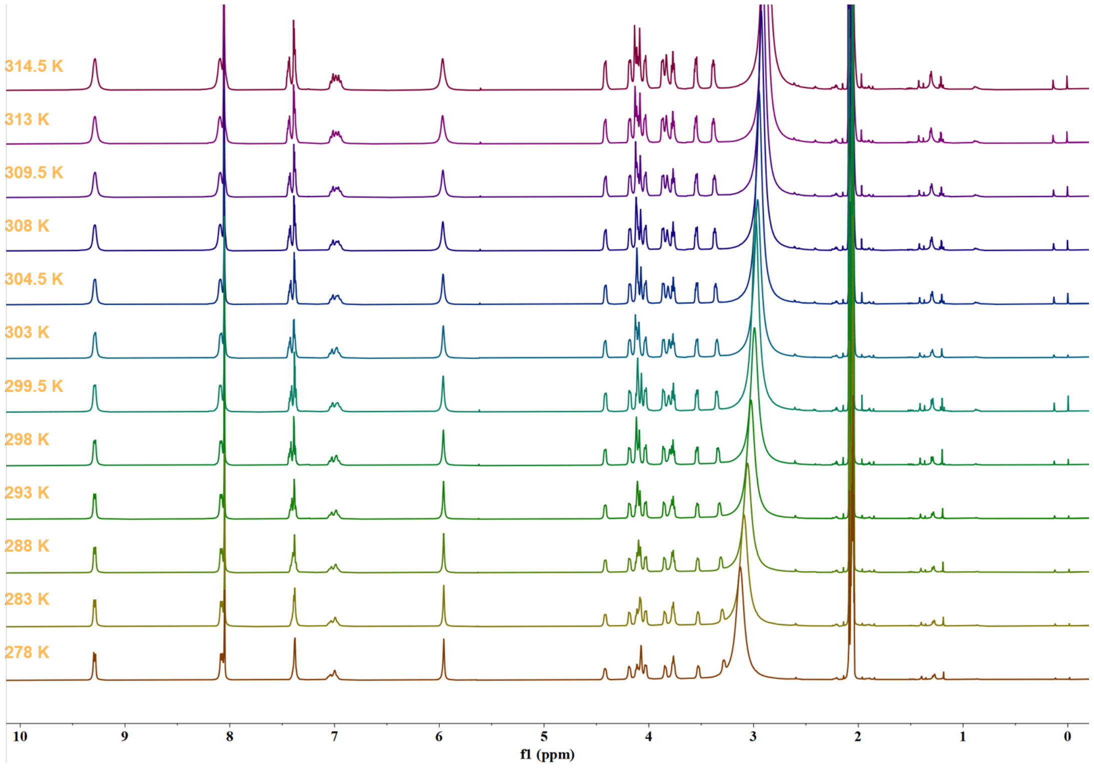


**Figure S31.** VT $^1H$ NMR spectra (400 MHz, $CD_3COCD_3$) of **1**-$H_2$·6$PF_6$.

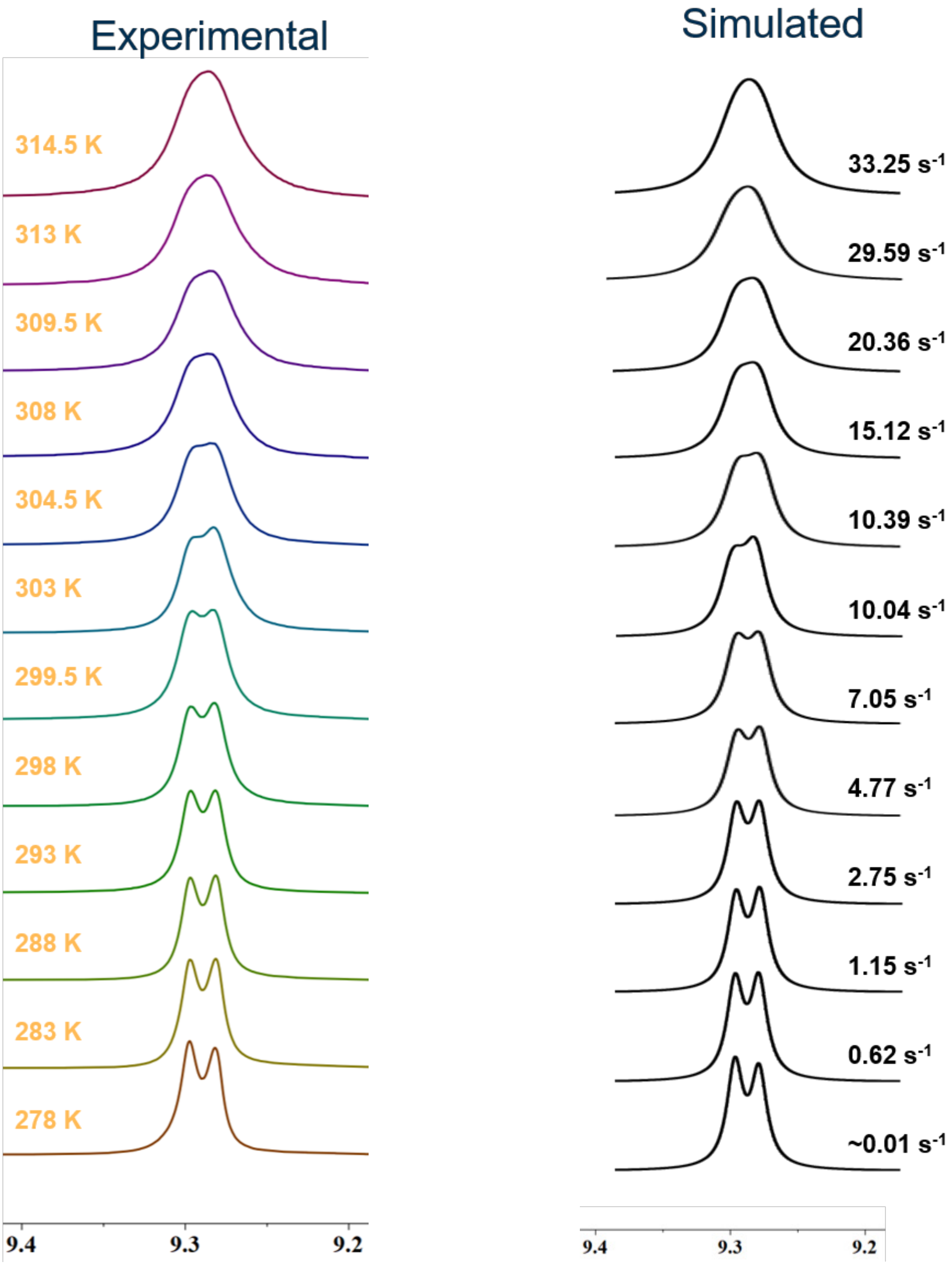


**Figure S32.** Experimental and simulated VT $^{1}$H NMR spectra of $H_t$ of **1**-$H_2$·6$PF_6$.

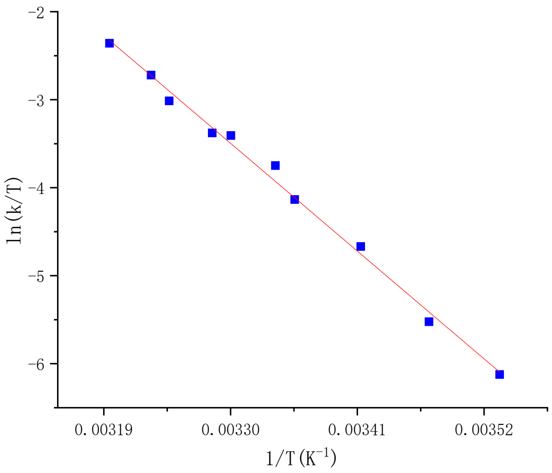


**Figure S33.** Eyring plot with trend line for the circumrotation rates of **1**-$H_2$·$6PF_6$.

$ln(k/T) = -\Delta H^{\neq}/RT + ln(k_B/h) + \Delta S^{\neq}/R$
$\Delta G^{\neq} = -RTln(K_{obs}h/k_BR)$

$\Delta H^{\neq}$ = 22.12 kcal·mol$^{-1}$
$\Delta S^{\neq}$ = 18.84 cal·mol$^{-1}$·K$^{-1}$
$\Delta G^{\neq}$ (298K) = 16.52 kcal·mol$^{-1}$

## 5.2 Determination of circumrotating rates of deprotonated catenane 1·$4PF_6$.

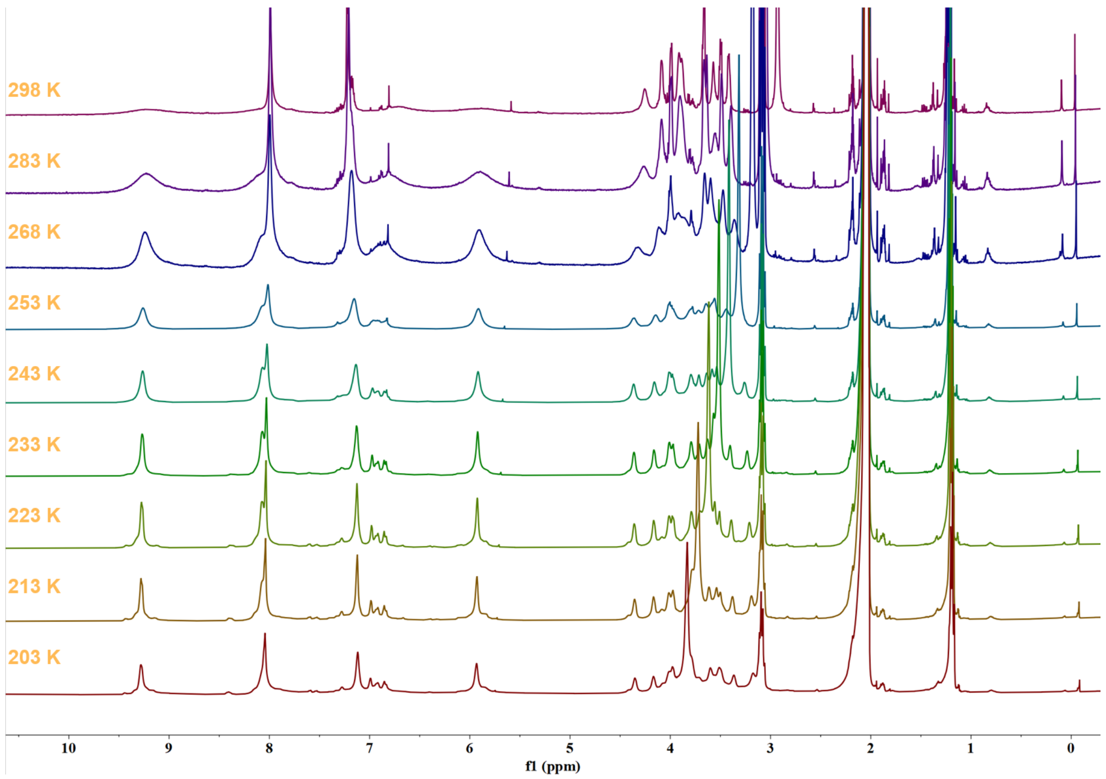


**Figure S34.** VT $^{1}H$ NMR spectra (400 MHz, $CD_3COCD_3$) of **1**·$4PF_6$.

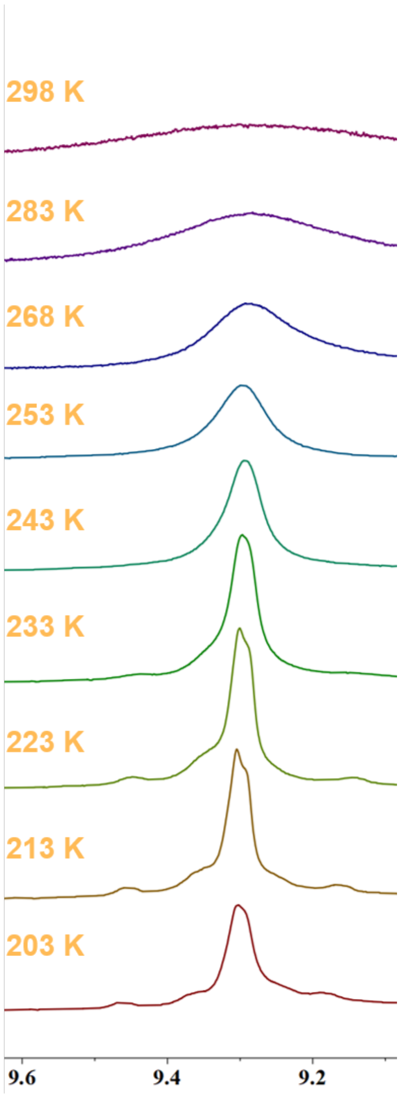


**Figure S35.** VT $^{1}H$ NMR spectra of $H_t$ of **1**·$4PF_6$.

# 6. UV-vis spectra of catenane by acid-base switching

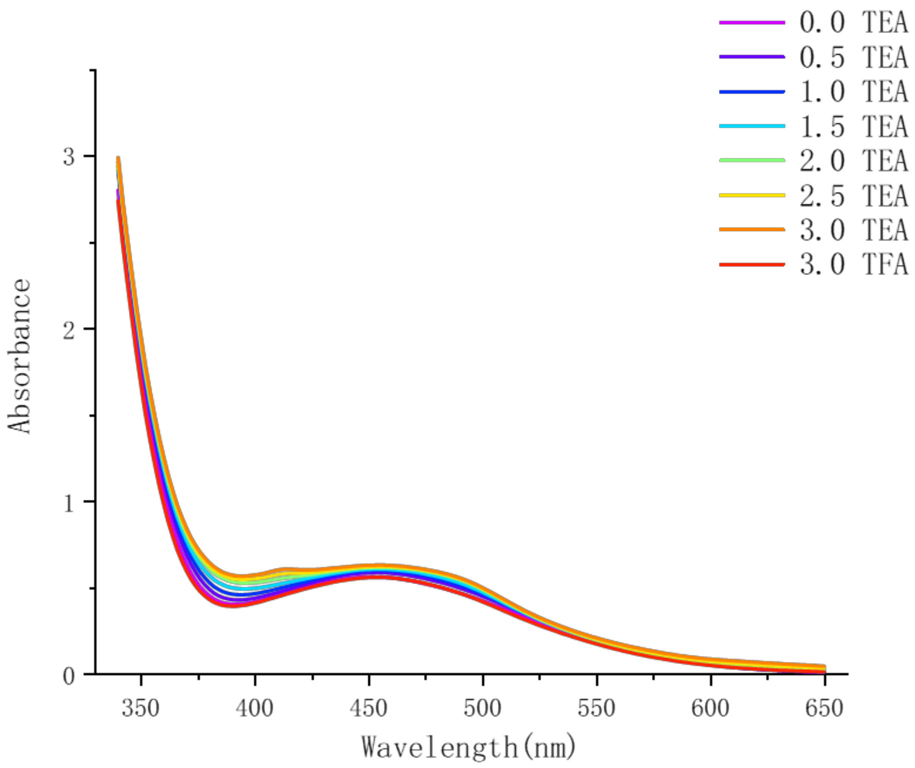


**Figure S36.** UV-vis absorption spectra of of **1**-$H_2 \cdot 6PF_6$ during the titration by TEA.

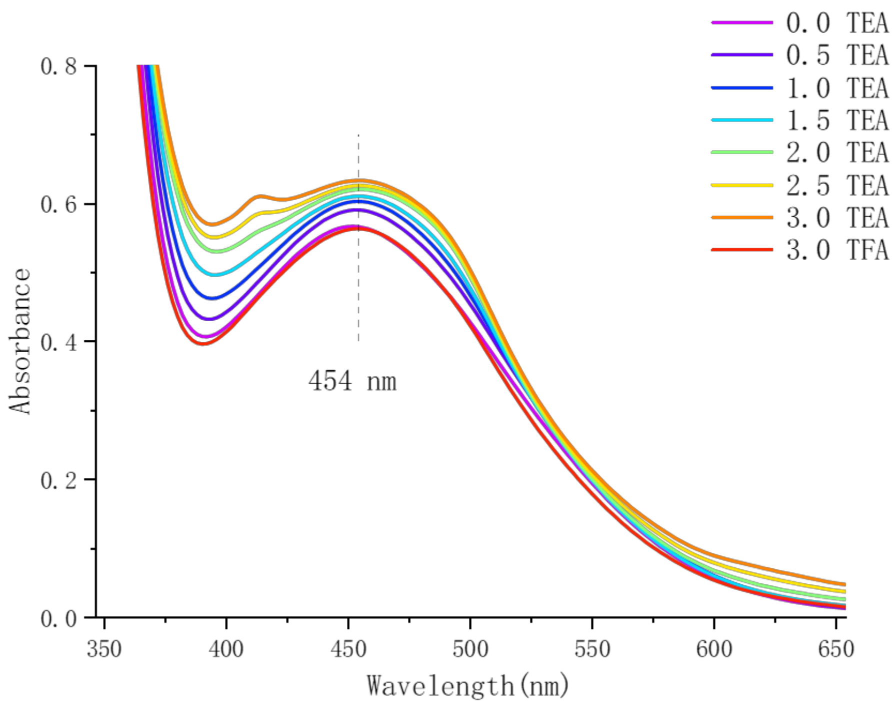


**Figure S37.** Partial UV-vis absorption spectra of of **1**-$H_2 \cdot 6PF_6$ during the titration by TEA.

# 7. The $^1$H NMR and $^{13}$C NMR spectra of the compounds

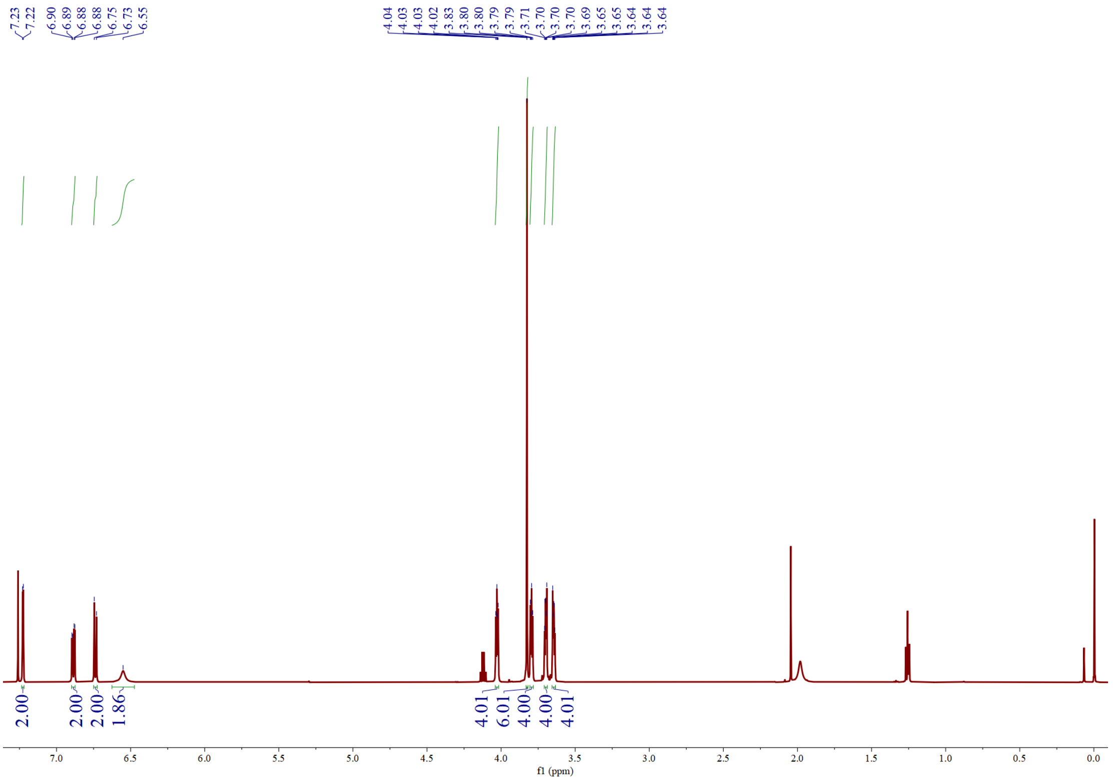

**Figure S38.** $^1$H NMR spectrum (600 MHz, $CDCl_3$, 298 K) of compound **8**.

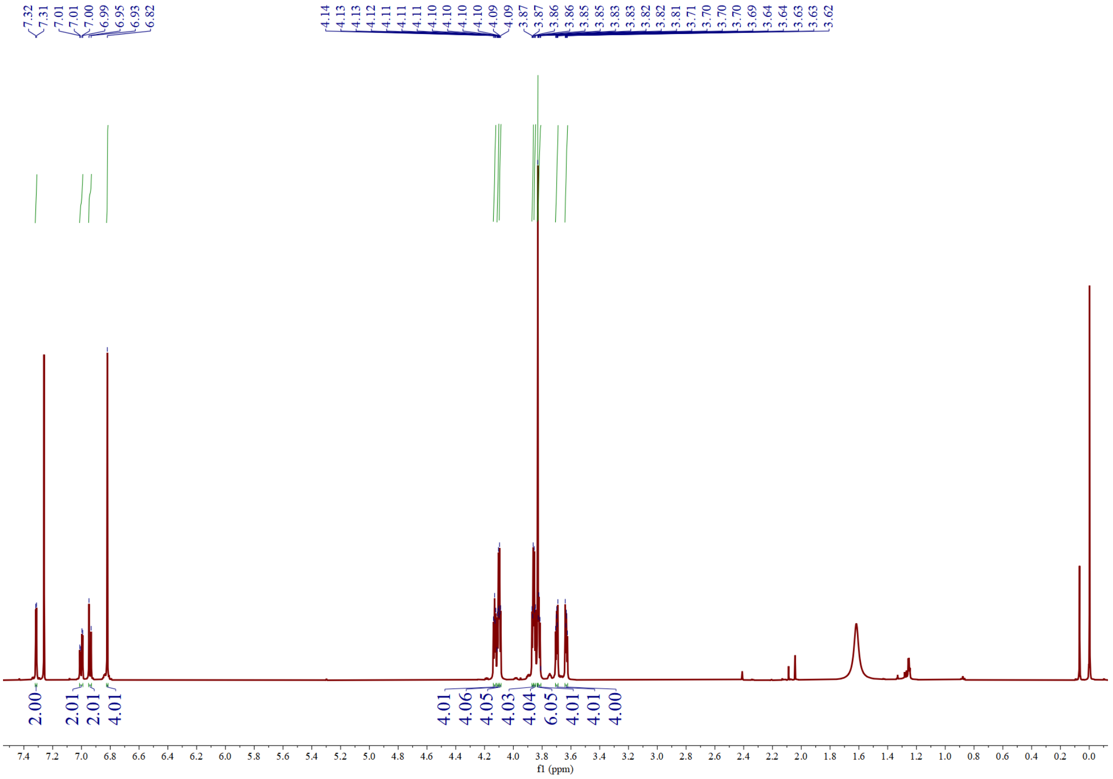

**Figure S39.** $^{1}H$ NMR spectrum (600 MHz, $CDCl_3$, 298 K) of compound **6**.

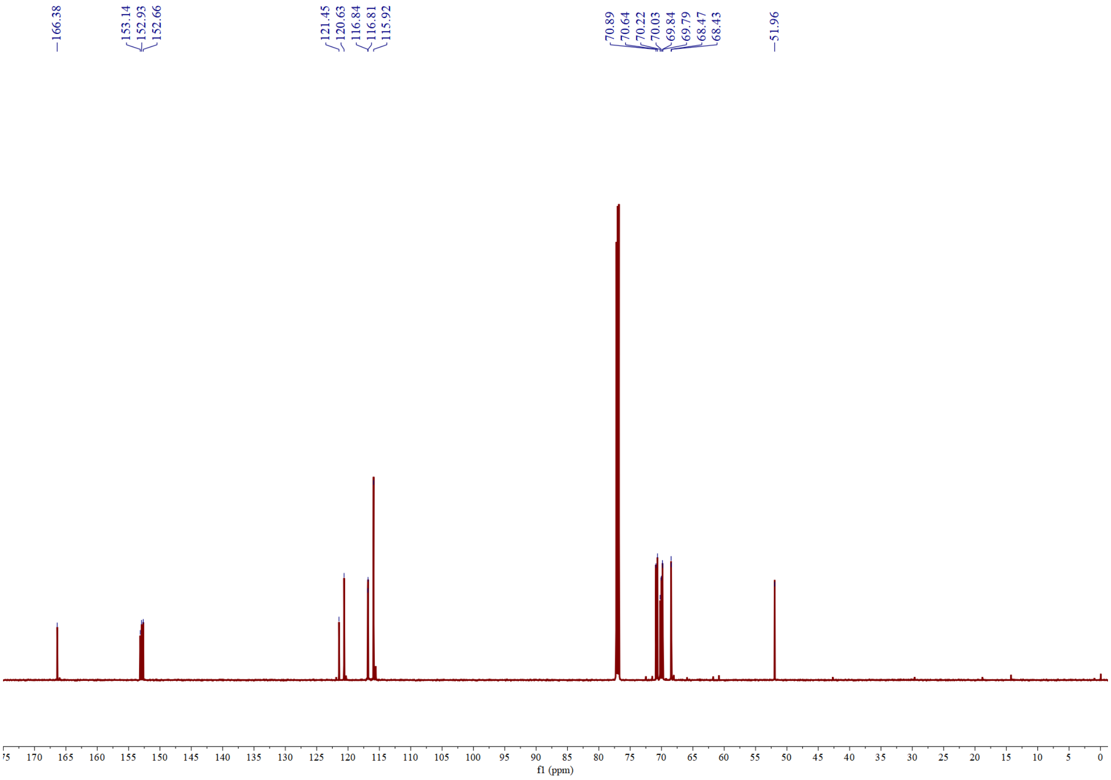


**Figure S40.** $^{13}C$ NMR spectrum (600 MHz, $CDCl_3$, 298 K) of compound **6**.

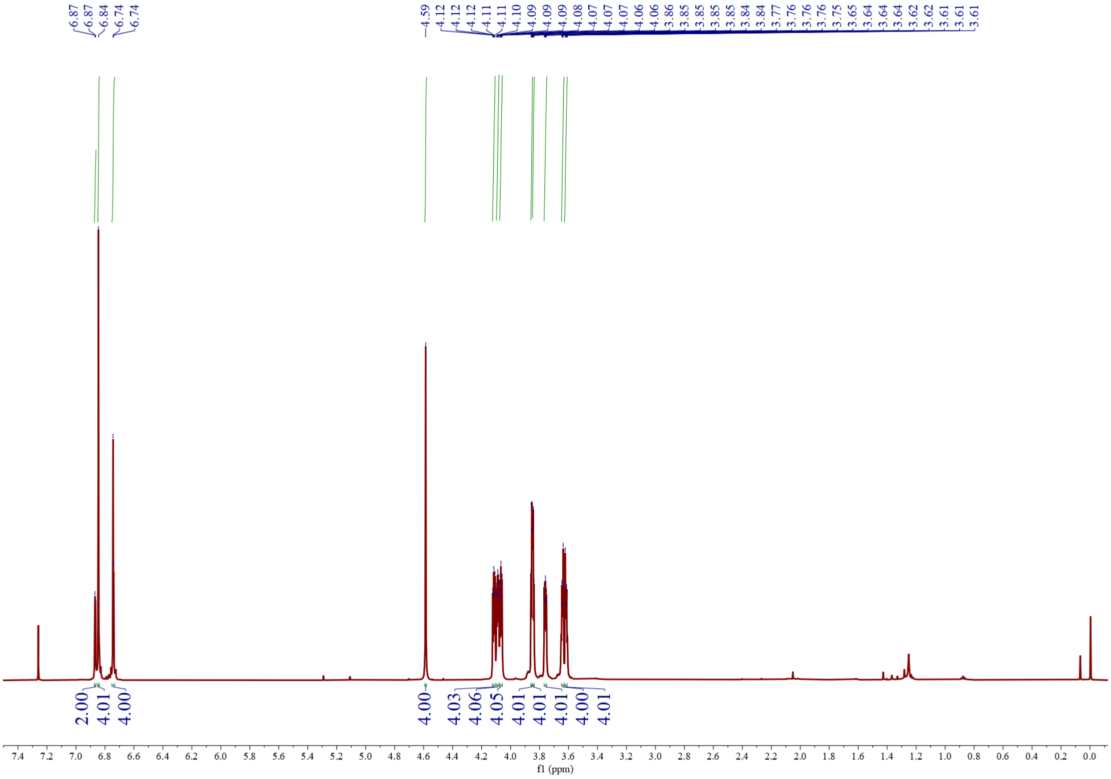


**Figure S41.** $^{1}H$ NMR spectrum (600 MHz, $CDCl_3$, 298 K) of compound **5**.

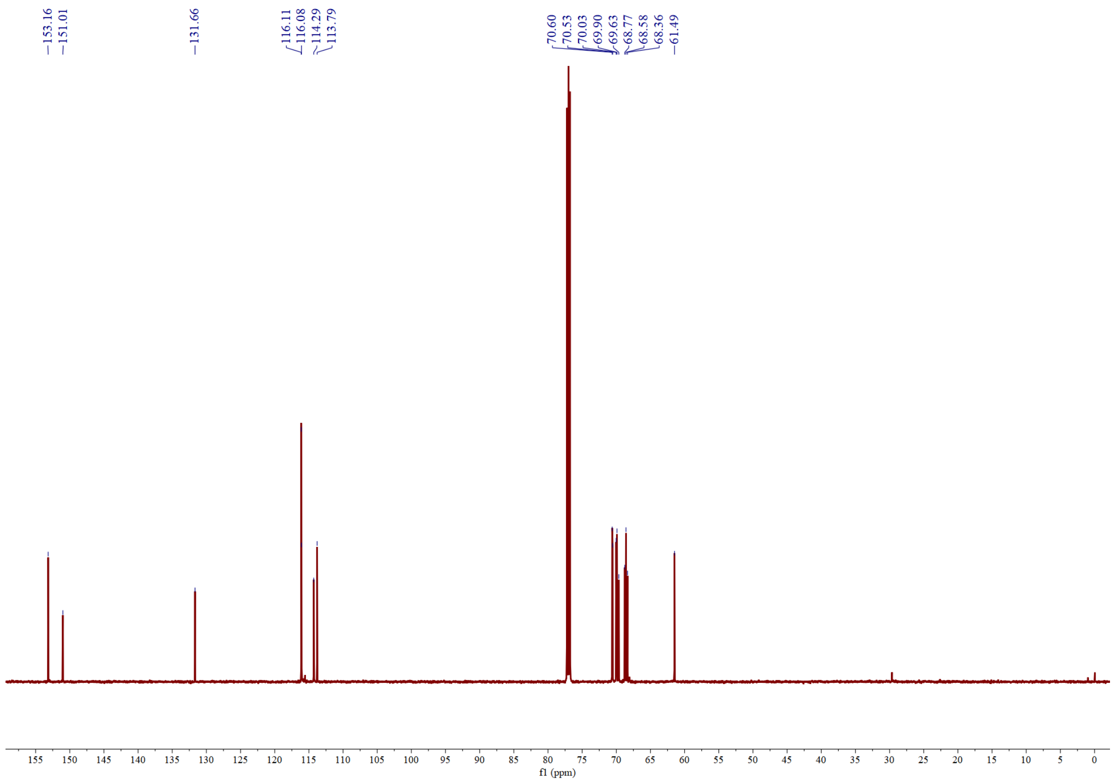

**Figure S42.** $^{13}C$ NMR spectrum (600 MHz, $CDCl_3$, 298 K) of compound **5**.

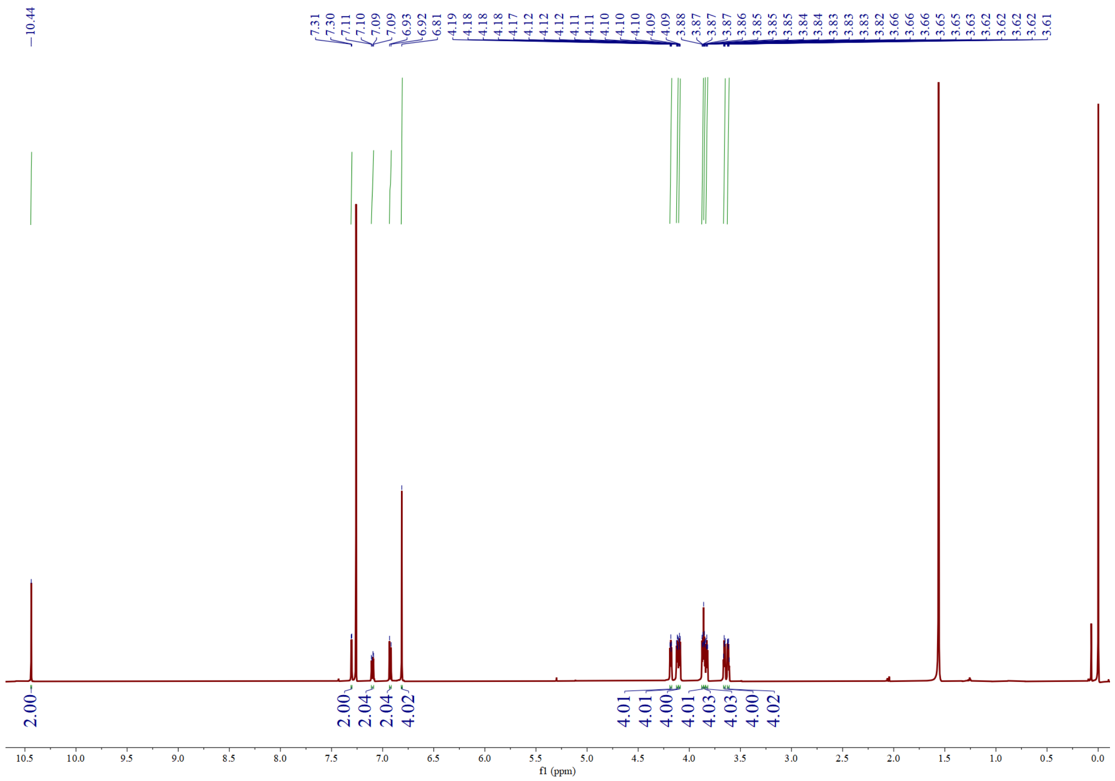

**Figure S43.** $^{1}H$ NMR spectrum (600 MHz, $CDCl_3$, 298 K) of compound **4**.

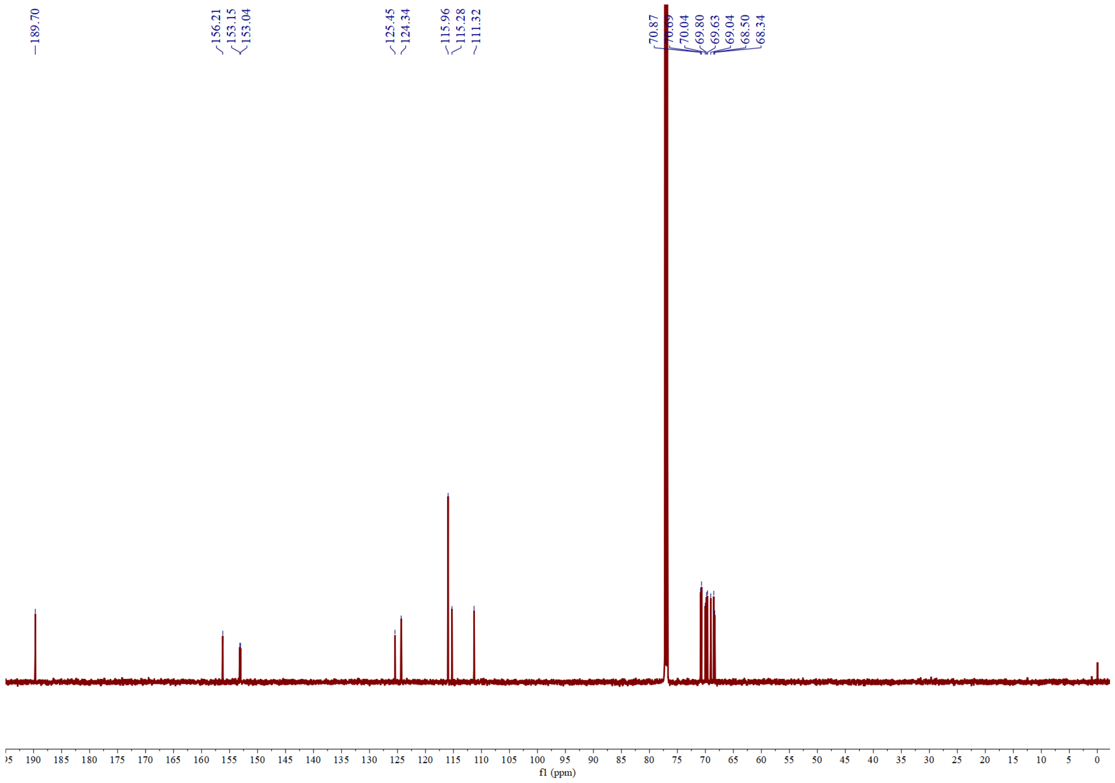

**Figure S44.** $^{13}C$ NMR spectrum (600 MHz, $CDCl_3$, 298 K) of compound **4**.

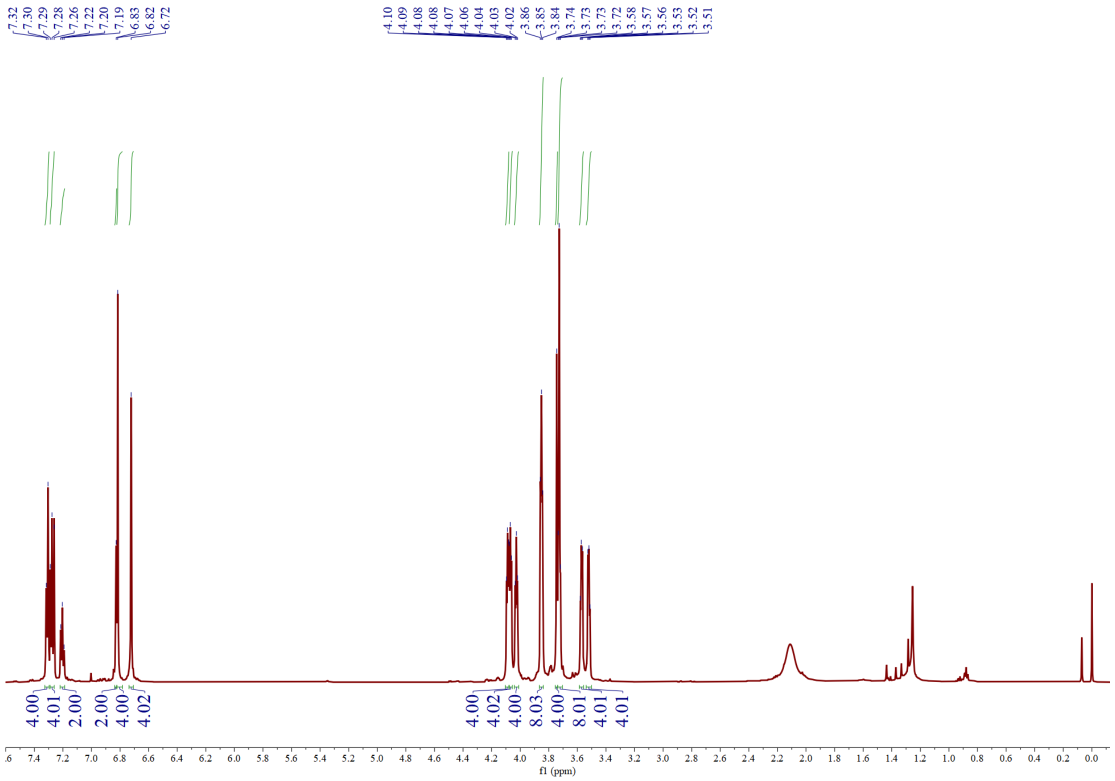

**Figure S45.** $^{1}H$ NMR spectrum (600 MHz, $CDCl_3$, 298 K) of compound **3**.

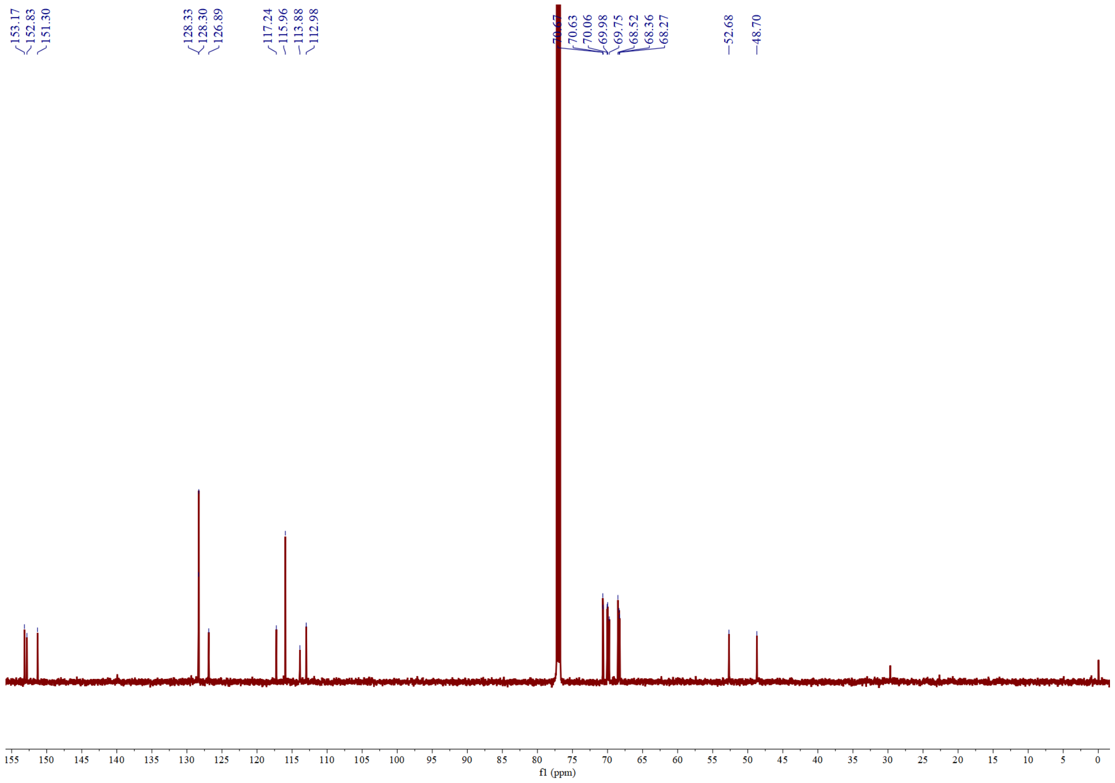

**Figure S46.** $^{13}C$ NMR spectrum (600 MHz, $CDCl_3$, 298 K) of compound **3**.

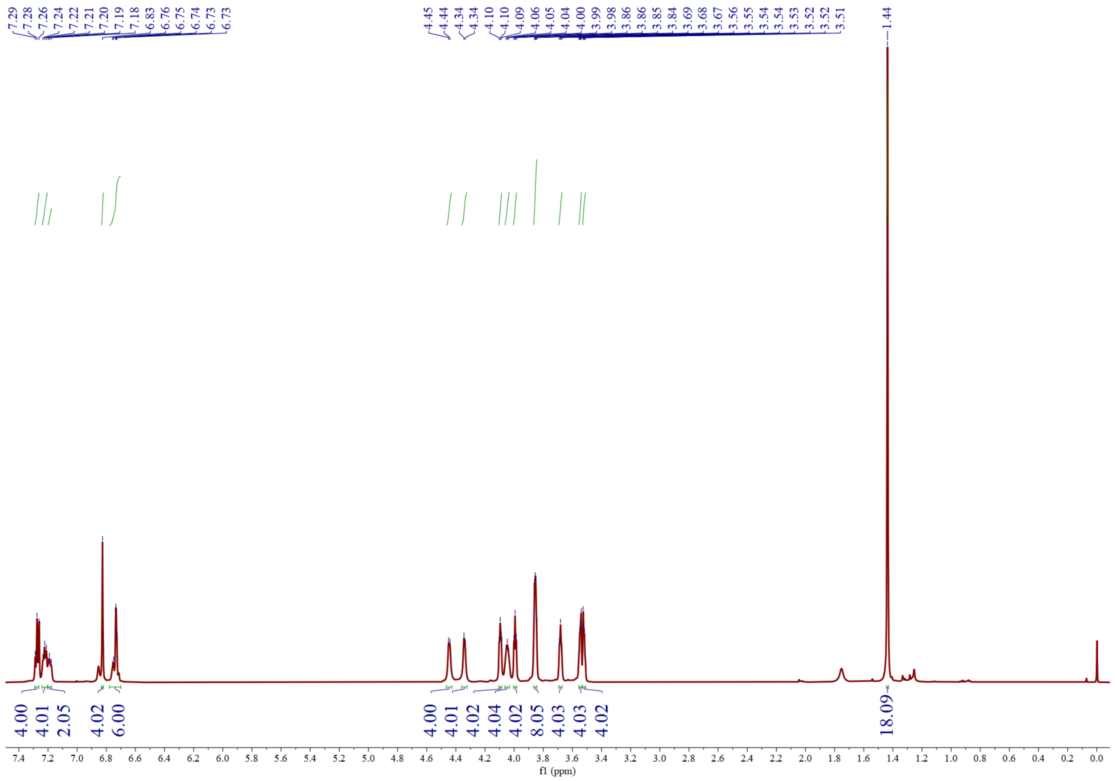

**Figure S47.** $^{1}H$ NMR spectrum (600 MHz, $CDCl_3$, 298 K) of compound **2**.

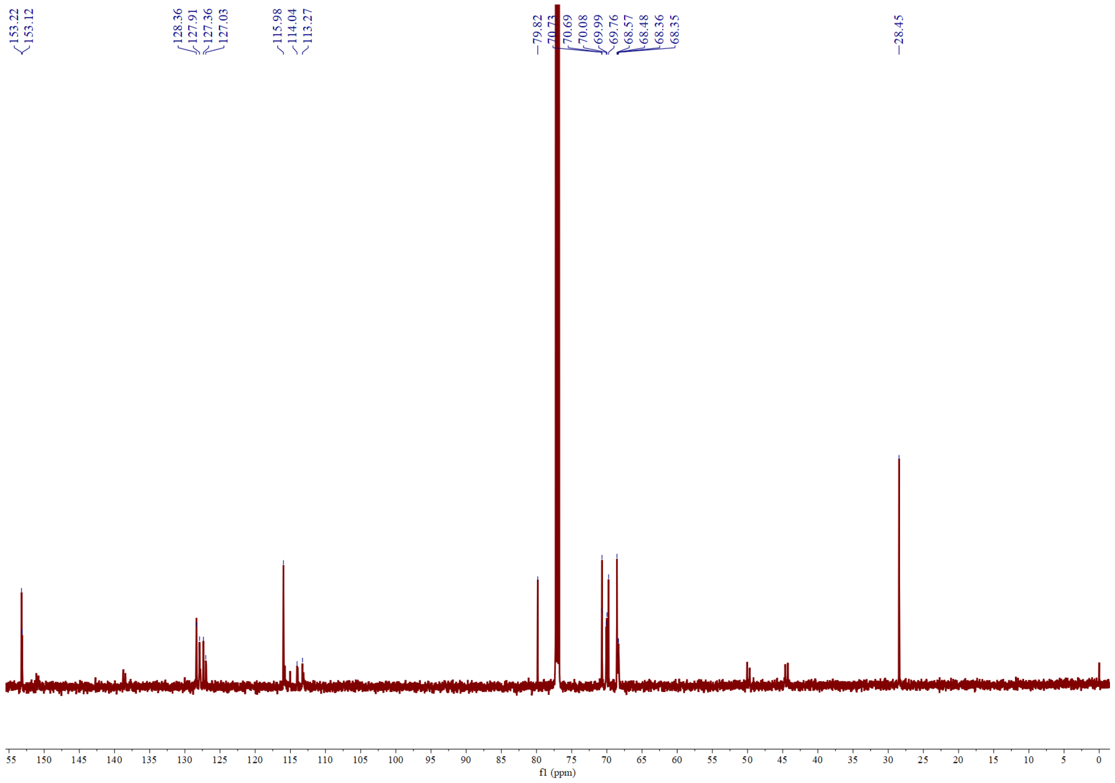

**Figure S48.** $^{13}C$ NMR spectrum (600 MHz, $CDCl_3$, 298 K) of compound **2**.

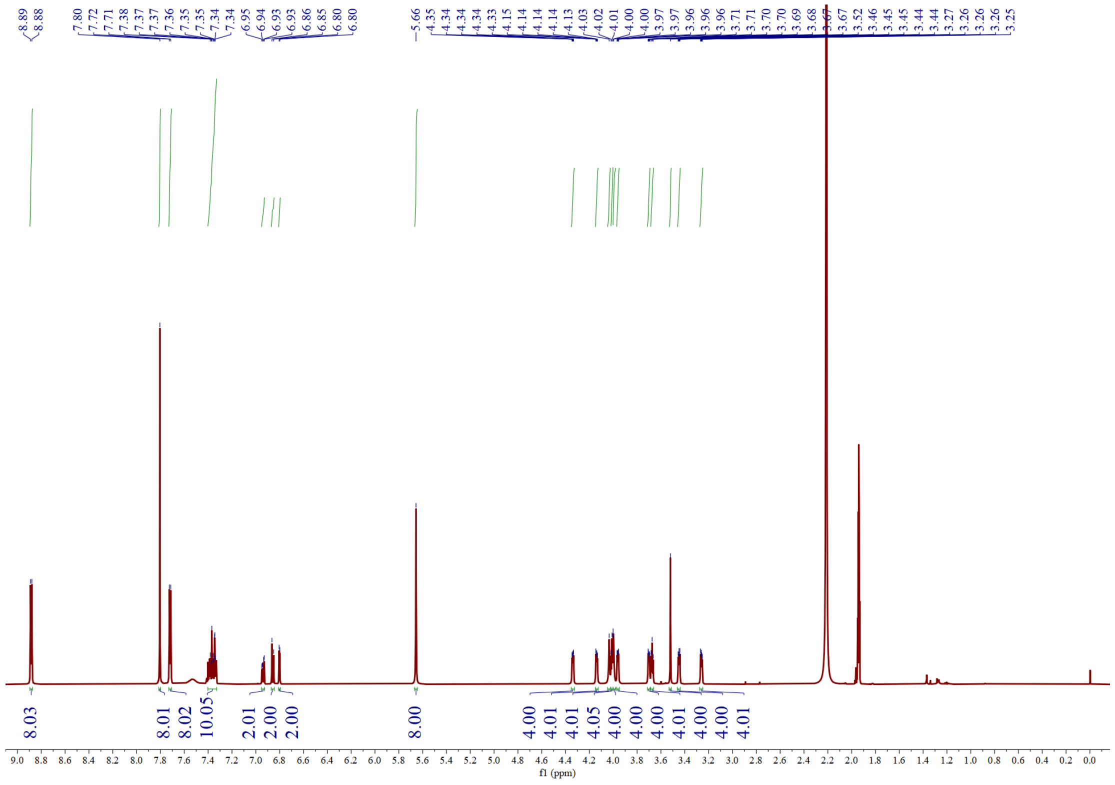

**Figure S49.** $^{1}H$ NMR spectrum (600 MHz, $CD_3CN$, 298 K) of [2]catenane **1**-$H_2 \cdot 6PF_6$.

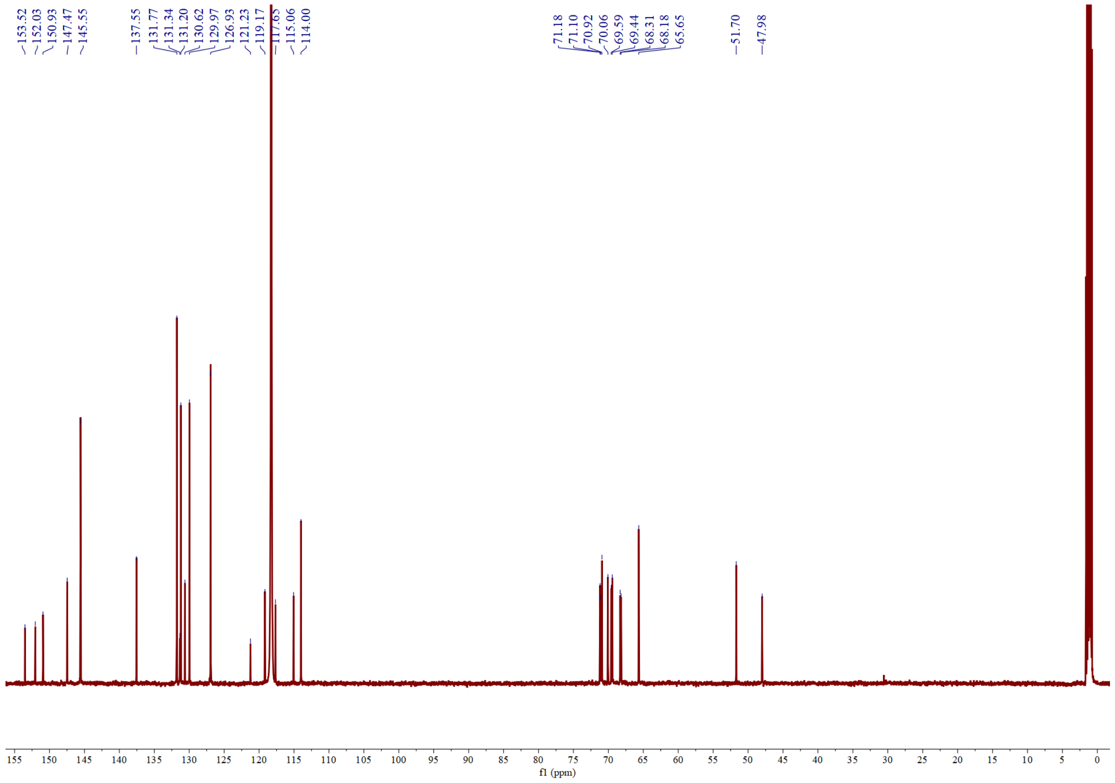


**Figure S50.** $^{13}C$ NMR spectrum (600 MHz, $CD_3CN$, 298 K) of [2]catenane **1**-$H_2 \cdot 6PF_6$.

# 8. The MS spectra of the compounds

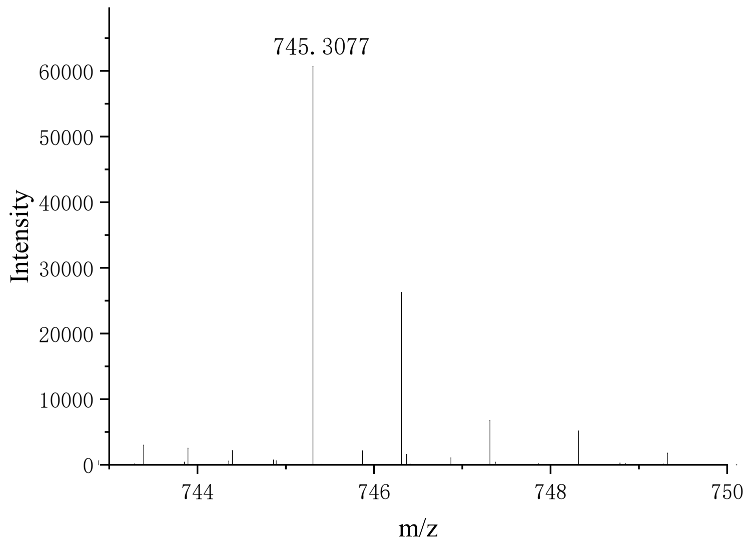


**Figure S51.** High resolution mass spectrum (ESI) of compound **6**.

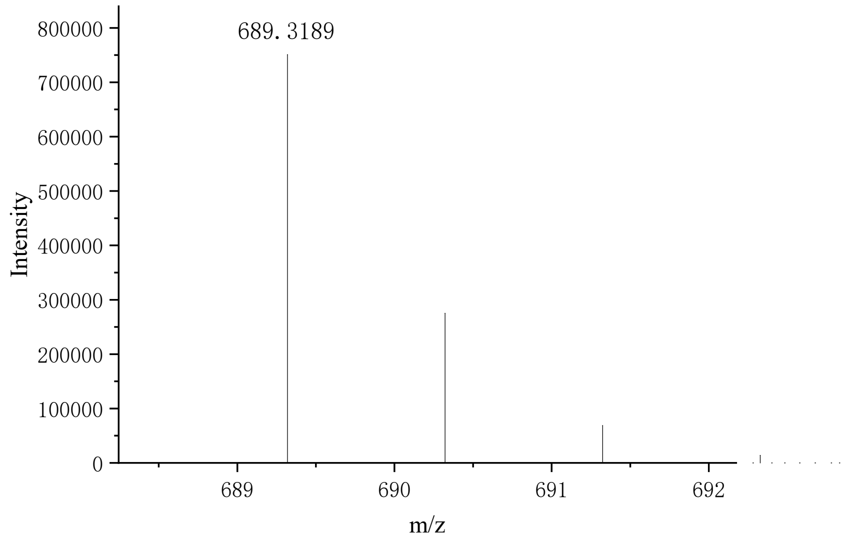


**Figure S52.** High resolution mass spectrum (ESI) of compound **5**.

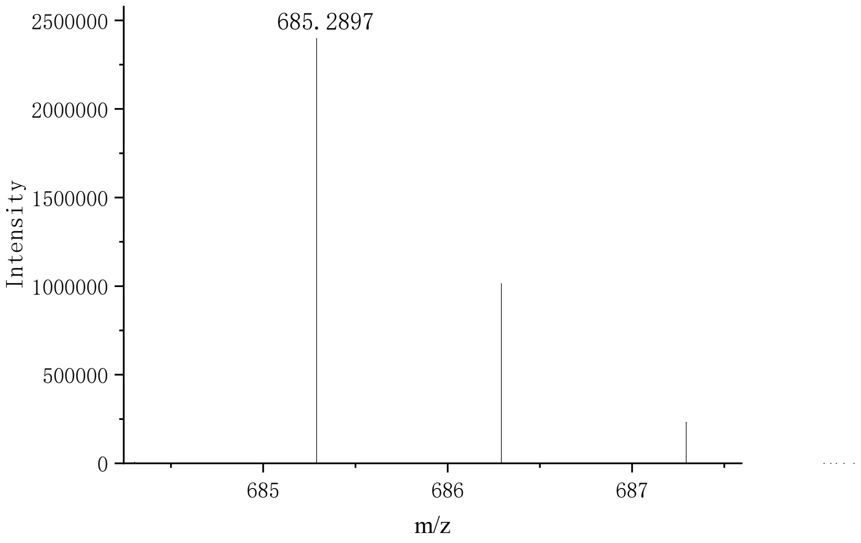


**Figure S53.** High resolution mass spectrum (ESI) of compound **4**.

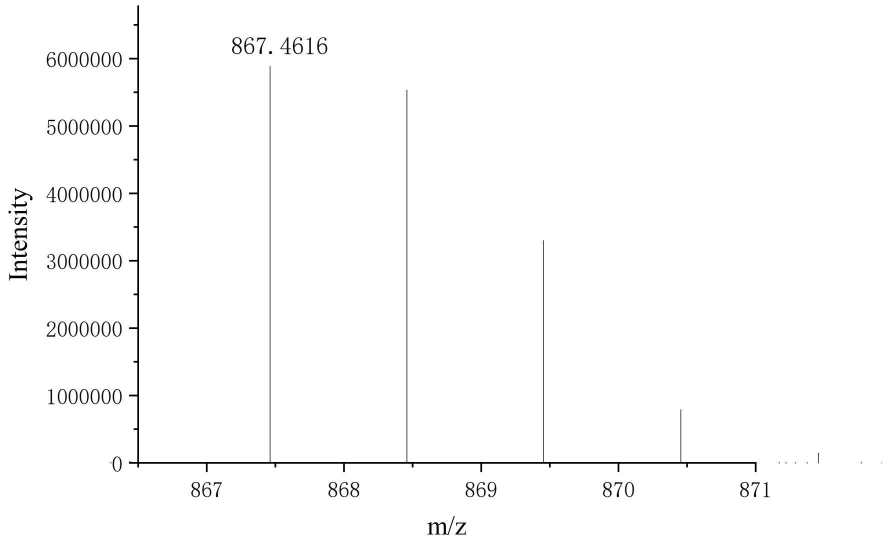


**Figure S54.** High resolution mass spectrum (ESI) of compound **3**.

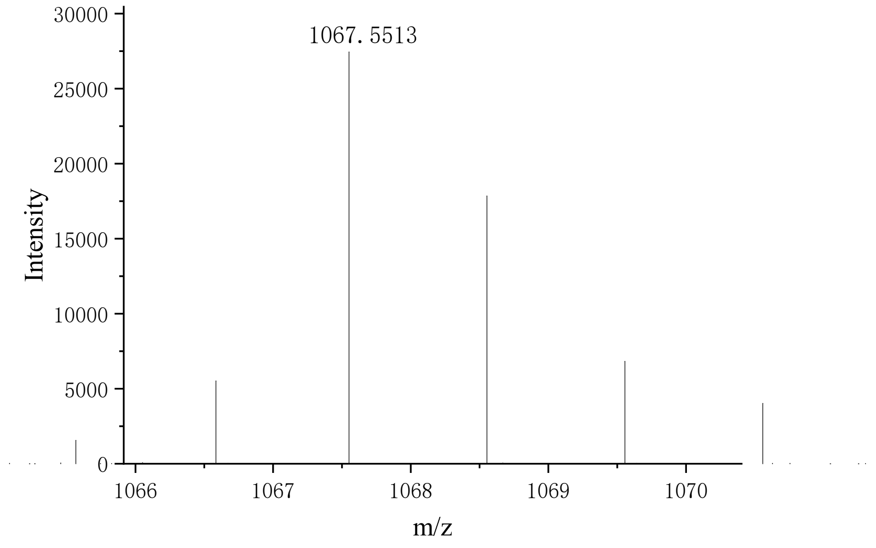


**Figure S55.** High resolution mass spectrum (ESI) of compound **2**.

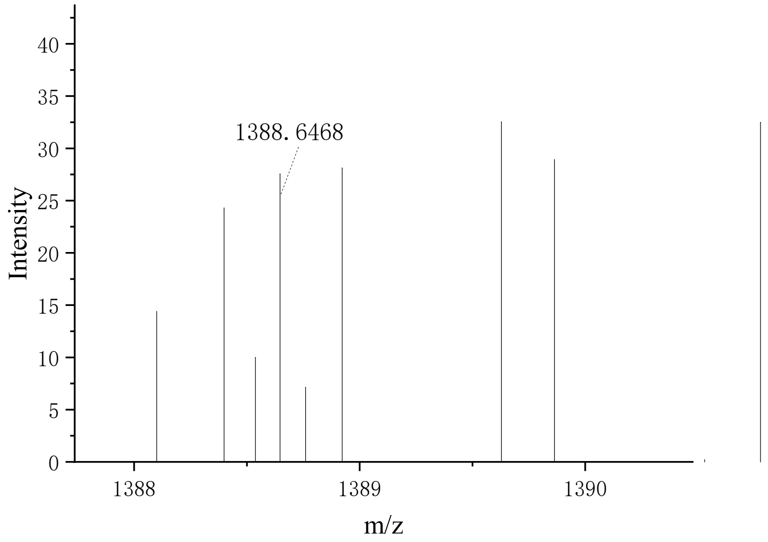


**Figure S56.** High resolution mass spectrum (ESI) of **1**-$H_2 \cdot 6PF_6$.

# 9. Reference


[1] V. N. Vukotic, K.-L. Zhu, G. Baggi, S. J. Loeb, *Angew. Chem. Int. Ed.* **2017**, *56*, 1-7.

[2] A. D. Bain, *Prog. Nucl. Magn. Reson. Spectrosc.* **2003**, *43*, 63–103.
